# The effect of Traditional Chinese Medicine on patients undergoing targeted therapy for primary liver cancer: a systematic review and meta-analysis

**DOI:** 10.3389/fonc.2025.1674965

**Published:** 2025-10-24

**Authors:** Hongting Yan, Yingjie Li, Bin Guo, Bing Yang, Dongxin Tang

**Affiliations:** ^1^ The First College of Clinical Medicine, Guizhou University of Traditional Chinese Medicine, Guiyang, Guizhou, China; ^2^ Guizhou University of Traditional Chinese Medicine, Guiyang, Guizhou, China; ^3^ Scientific Research and Education Department, Southern Medical University Hospital of Integrated Traditional Chinese and Western Medicine, Guangzhou, Guangdong, China; ^4^ Admissions Office, Guizhou University of Traditional Chinese Medicine, Guiyang, Guizhou, China; ^5^ Student Management Office, The First College of Clinical Medicine, Guizhou University of Traditional Chinese Medicine, Guiyang, Guizhou, China

**Keywords:** primary liver cancer, targeted therapy, traditional Chinese medicine, systematic review, meta-analysis

## Abstract

**Objective:**

Evaluate the therapeutic efficacy and safety of Traditional Chinese Medicine (TCM) in patients with primary liver cancer (PLC) receiving targeted therapy.

**Methods:**

We conducted a comprehensive search of databases. The search scope covered the period from the establishment of the databases to April 2025. We included 49 randomized controlled trials (RCTs) evaluating targeted therapy for primary liver cancer with TCM. Efficacy and safety outcomes were assessed using risk ratios (RR), standardized mean differences (SMD), and their 95% confidence intervals (CI).

**Results:**

Targeted therapy for liver cancer patients who received TCM treatment showed improvements in objective response rate (ORR) (RR, 1.49 [1.33-1.66], *P* < 0.0001), disease control rate (DCR) (RR, 1.32 [1.25,1.40], *P* < 0.0001), the 1-year survival (RR, 1.50 [1.20,1.88]; *P* = 0.0004) and Karnofsky Performance Status (KPS) (SMD, 1.34 [0.86,1.81]; *P* < 0.0001), and can reduce the incidence of adverse events, as well as to some extent decrease the production of tumor markers and related inflammatory factors.

**Conclusion:**

TCM enhances the efficacy and safety of targeted therapy in PLC, offering superior clinical outcomes with fewer adverse effects. These findings support its potential integration into standard treatment protocols.

**Systematic review registration:**

https://www.crd.york.ac.uk/prospero/, identifier CRD420251055085.

## 1 Introduction

The most recent findings from the International Agency for Research on Cancer (IARC) reveal that primary liver cancer (PLC) is the sixth most frequently diagnosed cancer worldwide and the third deadliest in terms of cancer fatalities (1). Its high incidence and mortality rates are primarily attributed to factors such as chronic hepatitis B virus (HBV) and hepatitis C virus (HCV) infections, aflatoxin exposure, and metabolic syndrome (2). As a prevalent gastrointestinal cancer, PLC poses significant threats to human health and remains a major therapeutic challenge. In clinical practice, the diagnosis and monitoring of PLC heavily rely on specific tumor markers, among which Alpha-fetoprotein (AFP) is the most widely used. AFP maintains normal or low levels throughout the life cycle but exhibits abnormal expression in PLC, becoming a risk factor. As a biologically active molecular protein in PLC, it participates in tumor growth, angiogenesis, and immune regulation, influencing pathways related to PLC cell proliferation and immune evasion. Elevated AFP levels are not only indicative of tumor presence but are also correlated with tumor burden, progression, and prognosis. Other emerging markers, such as Des-γ-carboxy prothrombin (DCP) and AFP-L3, are increasingly recognized for their diagnostic and prognostic value (3, 4). In addition to AFP, other laboratory parameters are critical for assessing liver function and overall patient status. These include liver enzymes (ALT, AST, ALP), measures of synthetic and metabolic function (ALB, TP, TBIL), and general tumor markers (CEA, CA125, CA19-9). Collectively, these biomarkers provide a comprehensive overview of liver health, synthetic capacity, and nutritional status, and are widely used to monitor disease progression and risk in primary liver cancer (5).Despite significant advances in screening and surveillance enabling earlier detection, diagnosis, and treatment of PLC, a substantial proportion (25-70%) of patients still present with advanced-stage disease at diagnosis and miss the chance for curative intervention (1, 6, 7). Although surgical resection, liver transplantation, and local therapies offer potentially curative options, their application remains limited to early-stage disease with small, localized tumors, while therapeutic efficacy is frequently compromised by the highly aggressive and metastatic nature of PLC (8, 9). Systemic therapies offer effective treatment options for advanced PLC patients with disease progression (10).

Tyrosine kinase inhibitors (TKIs), as the backbone of systemic therapy for advanced PLC, have ushered in a new era of systemic treatment. Sorafenib, indicated for unresectable PLC, remained the only systemic therapy with proven survival benefits in advanced-stage patients for many years. Lenvatinib, another TKI, has demonstrated non-inferior survival outcomes to sorafenib, establishing both agents as first-line therapies for advanced PLC, which effectively delay disease progression (8, 11, 12). However, their therapeutic efficacy remains constrained by adverse effects and acquired resistance, representing persistent challenges in targeted therapy. In the REFLECT trial (13), lenvatinib-treated patients exhibited higher incidences of hypertension and proteinuria, whereas sorafenib-treated patients experienced more frequent cutaneous reactions and diarrhea. These distinct toxicity profiles similarly compromised quality-of-life (QoL) metrics in both treatment arms. Recent years have witnessed significant advances beyond targeted therapy, with emerging evidence demonstrating the efficacy of both immunotherapy monotherapy (14) and immune-targeted combinations (15), which have been incorporated into the NCCN (2024) guidelines. While systemic therapies have markedly improved survival outcomes and disease control rates in advanced PLC patients, their clinical benefits remain constrained by intrinsic resistance, treatment-related toxicities, and persistently poor overall prognosis. These challenges have driven the development of integrated therapeutic strategies combining targeted agents with other modalities—including immunotherapy, local therapies, and traditional medicines—aiming to enhance treatment efficacy while mitigating adverse effects.

With a history of thousands of years in clinical practice, Traditional Chinese Medicine (TCM) has demonstrated its rationale and efficacy in managing complex diseases. A growing body of contemporary research now suggests that TCM may offer novel solutions to modern challenges in cancer treatment (16). TCM theory posits that the pathogenesis of liver cancer involves a complex interplay of “Qi stagnation,” “Blood stasis,” and “Toxic accumulation.” According to its theory, TCM proposes that the overall treatment principles are to strengthen the body’s resistance and eliminate evil. As an important method of cancer treatment in China, TCM, as natural substances, has long been considered to have the advantages of multi-pathway and multi-targets in tumor treatment and exerts multifaceted pharmacological active effects through comprehensive regulation, showing potential value in alleviating adverse effects, reversing drug resistance, inhibiting metastasis, regulating tumor immunity and improving therapeutic efficacy (17, 18). The holistic concept of Chinese medicine coincides with the systematic treatment of tumors as a systemic disease, and the idea of “living with the tumor” not only seeks to improve the progression of the disease, but also focuses on improving the quality of life of the patient. Clinically, TCM is often used alongside conventional cancer treatments with the goals of ameliorating side effects, improving physical performance, and potentially sensitizing tumors to targeted drugs. Accumulating evidence confirms that TCM can significantly inhibit the migration, invasion, and proliferation of liver cancer cells, thereby contributing to prolonged overall survival (OS) and progression-free survival (PFS) in patients with PLC. Furthermore, Chinese herbal medicine has been shown to mitigate hepatotoxicity and gastrointestinal toxicity, potentially through the downregulation of a spectrum of inflammation-related chemokines (19). It is precisely these characteristics that make TCM a suitable candidate for combination therapy with other therapies, thereby achieving synergistic therapeutic effects against cancer.

Despite growing interest and clinical application, the evidence supporting the combination of TCM with targeted therapy for PLC remains fragmented and has not been comprehensively synthesized. Previous systematic reviews have often been limited by small sample sizes, a focus on a single TCM formula, or a lack of quantitative meta-analysis. Moreover, no rigorous quantitative synthesis of the efficacy and safety of this integrated therapy has yet been conducted in the existing literature. The primary objective of this systematic review and meta-analysis is to determine whether TCM, when combined with targeted therapy, improves survival benefits and safety outcomes for patients with PLC receiving targeted therapy compared to targeted therapy alone. This will be achieved by critically evaluating and statistically synthesizing existing evidence from randomized controlled trials (RCTs). By doing so, we aim to enhance clinical efficacy and reduce adverse events in patients with PLC, thereby improving their overall treatment outcomes.

## 2 Methods

The systematic research program is registered in PROSPERO under ID number CRD420251055085. The study adheres strictly to the Preferred Reporting Items for Systematic Reviews and Meta-Analyses(PRISMA) protocol, ensuring methodological transparency and comprehensive reporting standards throughout the investigation (20).

### 2.1 Eligibility criteria

Inclusion criteria followed the PICOS framework for condition identification:

Participants (P): all included cases must be confirmed as PLC patients after pathological/histological or imaging diagnosis. There are no gender, race, or country restrictions.
Intervention (I): The intervention cohort of this study receives TCM in combination with targeted therapeutic agents; there is no restriction on the combination of targeted therapeutic regimens, but the herbal medicines that must be used are all taken orally.
Comparison (C): In the control cohort, inclusion of treatment regimens receiving targeted therapeutic agents based on targeted therapies; there is no restriction on the use of other therapeutic regimens alone or in conjunction.
Outcome (O): To evaluate the efficacy and safety of oral herbal medicines with targeted therapy.
Study design: This systematic study used an RCT for analysis design.

The exclusion criteria applied were as follows: (1) incomplete or missing data, which made it impossible to extract data; (2) duplicate publications or duplicate data; (3) non-Chinese and English literature; (4) unavailability of full text; and (5) absence of important outcome indicators.

### 2.2 Outcome indicator

Primary outcome: Assessment criteria were based on World Health Organization (WHO) and Response Evaluation Criteria in Solid Tumors (RECIST) guidelines. Based on remission (CR), partial remission (PR), stable remission (SD), progressive remission (PD), and summarized as overall remission rate (ORR) and disease control rate (DCR).

Secondary outcomes: These comprised Karnofsky Performance Status (KPS) scores and 1-year survival. Tumor makers, liver function (ALT, AST, ALB, TBIL), immunologic profiles (CD3+/CD4+/CD8+ T cells, CD4+/CD8+ ratio, NK cells), and safety parameters were analyzed. Serum TNF-α, IL-6, and VEGF levels were also quantified.

### 2.3 Search strategy and study selection

Two researchers independently conducted a comprehensive literature search across international databases (Cochrane Library, PubMed, EMBASE, and Web of Science) and Chinese databases (CBM, CNKI, and Wanfang) to identify eligible studies published from database inception to April 2025. A total of 3,226 potentially relevant articles were initially identified. The search strategy was structured into four key domains: Traditional Chinese Medicine (TCM), including terms such as “herbal medicine”, “decoction”, “capsule”, and “formula”; Liver cancer, covering “hepatocellular carcinoma”, “liver neoplasms”, and “hepatic malignancies”; Targeted therapy, incorporating agents like “sorafenib” and “lenvatinib”; Randomized controlled trials (RCTs), using keywords such as “randomized,” “RCT,” and “clinical trial.” Chinese databases were searched using corresponding Chinese keywords. All domain-specific terms were combined using Boolean operators for the initial search (Full search strategy is available in **Supplementary Material 1**). Two assessors independently reviewed titles and abstracts for initial eligibility. Full-text articles were evaluated for final inclusion, with discrepancies resolved by consulting a third researcher.

### 2.4 Data extraction

The study collected information on the included trials and participants, including authors of literature and year, the type of research protocol, the average age of subjects, the sex composition ratio, sample size, details of oral TCM interventions (dosage and duration), specifications of targeted therapy regimens (dosage and cycles), additional combined treatments (dosage and cycles), and outcome measures. Any discrepancies in data extraction were resolved through consensus discussions among researchers.

### 2.5 Risk of bias assessment

The methodological rigor of the included studies was evaluated using the Cochrane Risk of Bias tool in RevMan 5.4, which examined seven key areas: randomization, concealment of allocation, blinding of participants and researchers, blinding of outcome assessors, handling of incomplete data, potential selective reporting, and other sources of bias. Each paper was then classified as having a low, high, or uncertain risk of bias based on these criteria.

### 2.6 Statistical analysis

Statistical evaluation was performed with RevMan 5.4 and R 4.4.1. Treatment effects were quantified as RR/SMD with 95% CIs (*P* < 0.05). Heterogeneity was assessed via the Q test/I^2^ statistics (*P* ≤ 0.1 or I^2^ > 50% indicating significance), determining fixed/random-effects model application. Publication bias (≥ 10 studies) was assessed via funnel plots, while sensitivity analyses examined individual study impacts. Subgroups were analyzed according to whether the combination treatment involved other therapies.

## 3 Results

### 3.1 Literature search study characteristics

**Figure 1:**
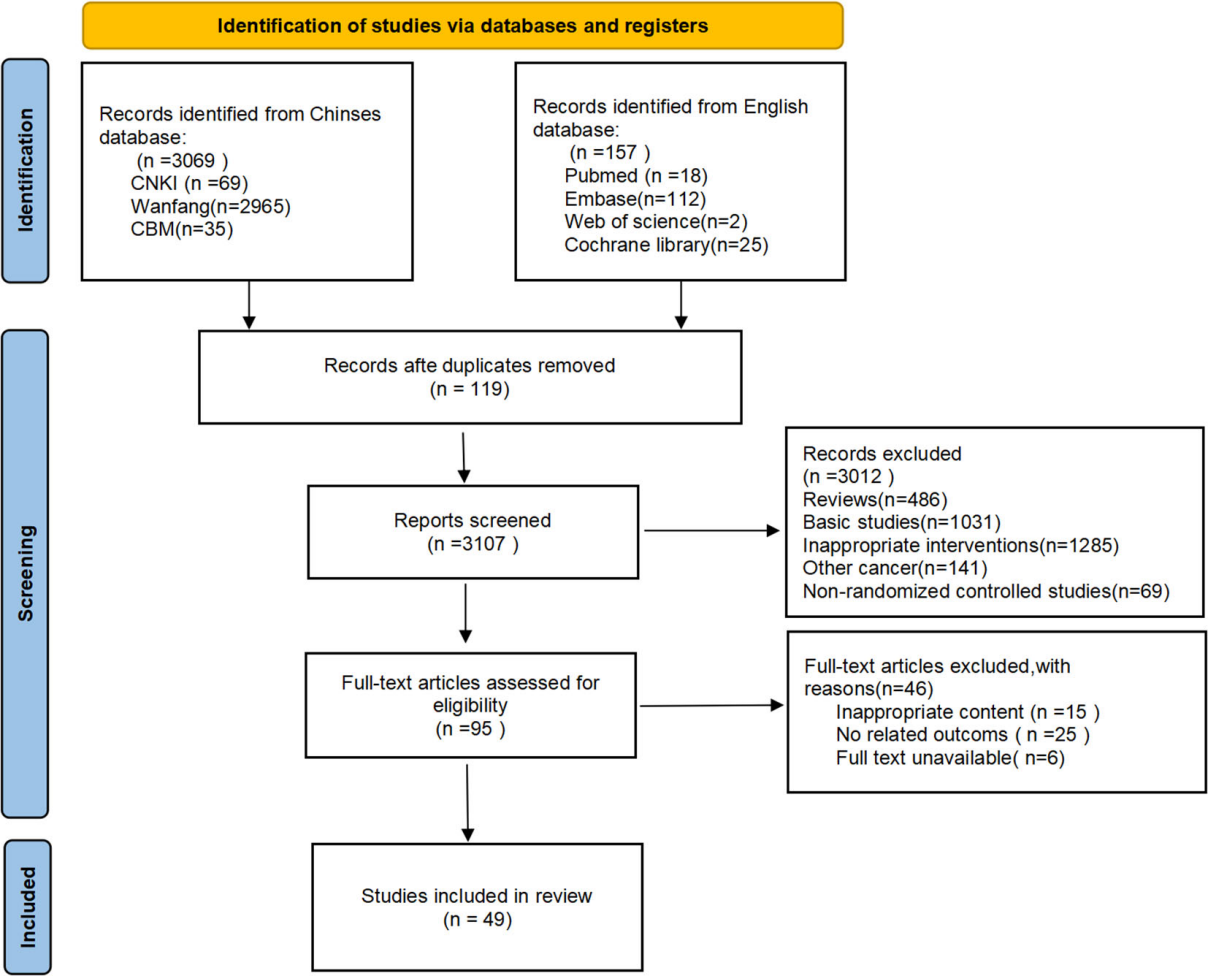
PRISMA flow diagram.

From an initial 3,226 records, 3,107 non-duplicate studies were screened, yielding 51 full-text articles. Final analysis included 49 China-based RCTs evaluating oral TCM. See **Figure 1** for the selection flowchart and **Supplementary Table 1** for study characteristics.

### 3.2 Methodological biases

**Figure 2:**
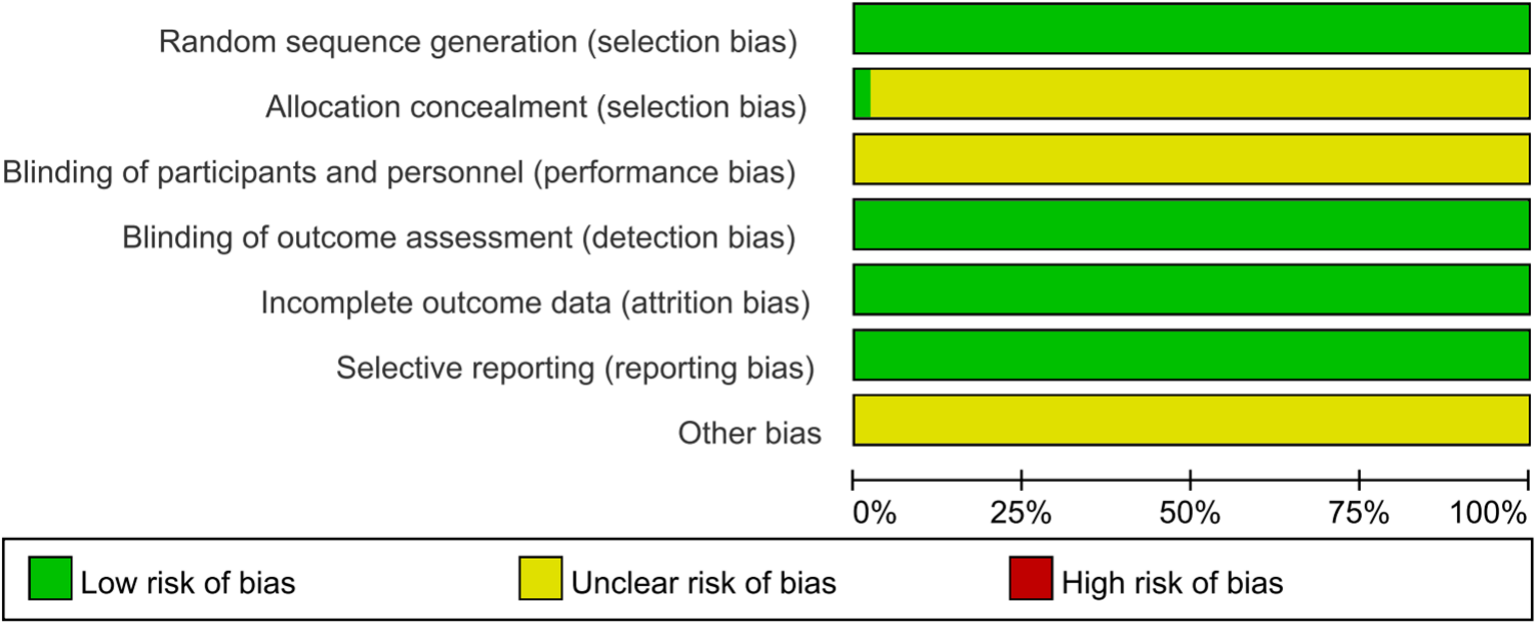
Risk of bias visualization analysis. The results of the researchers’ comprehensive evaluation of each risk of bias dimension in all included literature, presented in percentage form.

**Figure 3:**
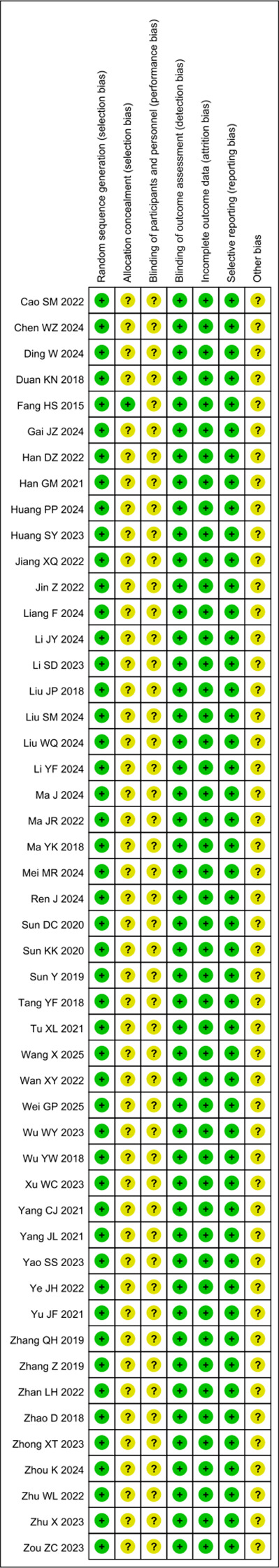
Risk of bias assessment results. Systematic evaluation of the risk of bias indicators by the investigators for each of the included studies in the literature.

Bias risk was assessed for all 49 studies. While all studies clearly described random allocation methods, the concealment of allocation remained unclear. None of the studies explicitly stated whether participants and investigators were blinded to group assignment. The outcome data were complete, with no sign of selective reporting observed across the studies. Additionally, no other potential sources of bias were identified (**Figures 2**, **3**).

### 3.3 Outcome indicator

#### 3.3.1 Tumor response

**Figure 4:**
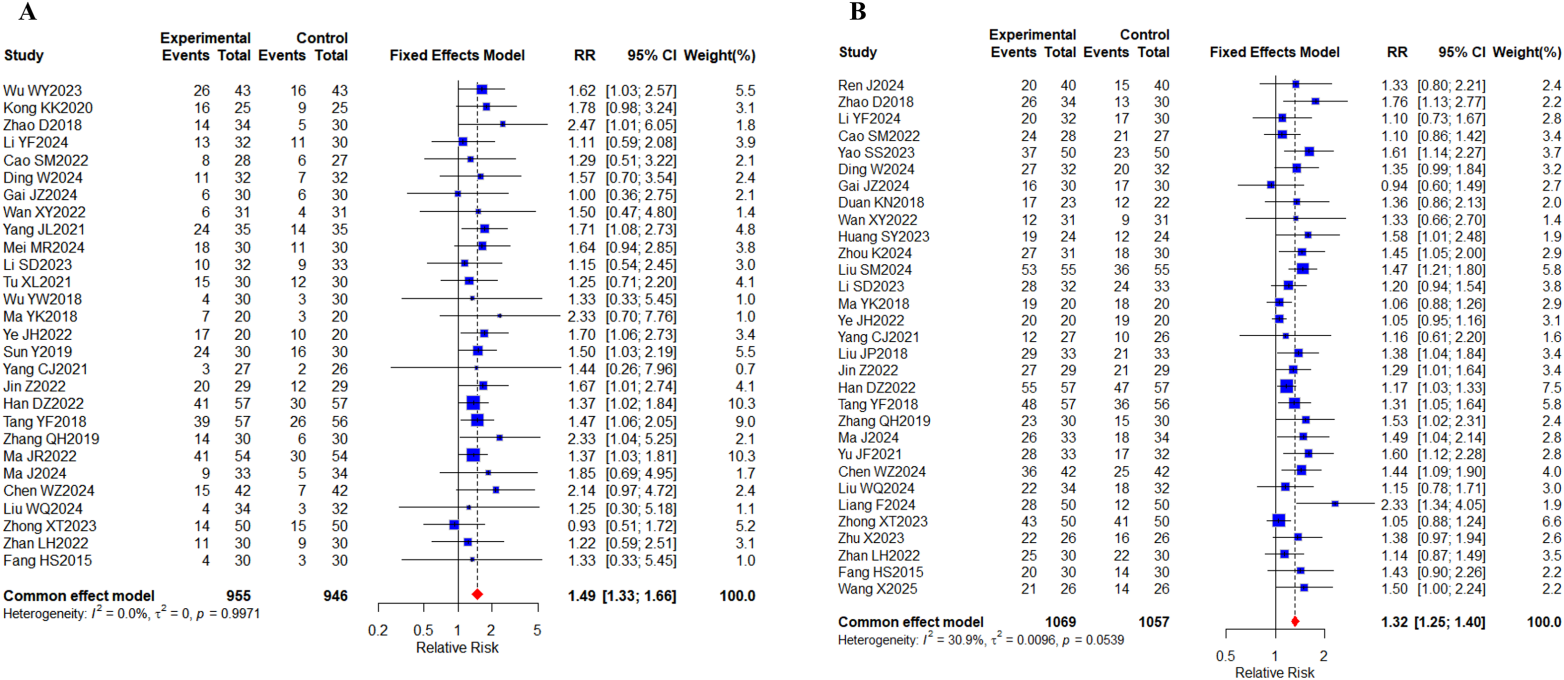
Tumor response. **(A)** Forest plot of ORR meta-analysis results; **(B)** Forest plot of DCR meta-analysis results.

World Health Organization (WHO) criteria (21) or Response Evaluation Criteria in Solid Tumors (RECIST) guidelines (22), both of which provide reference standards for monitoring the diagnosis and treatment of malignant tumors and evaluating their efficacy. Based on the criteria provided above, a total of 49 studies (23–71) were conducted, of which 28 (25, 26, 28–30, 32, 33, 35, 39, 40, 43, 45–50, 53–58, 60, 61, 63, 66, 70) included 1901 cases reporting ORR and 31 (24, 28–36, 38, 41, 43, 47, 48, 50, 52–56, 58–63, 65, 66, 70, 71) studies included 2126 cases reporting DCR (**Figures 4A, B**). The results of the systematic analysis showed no heterogeneity (ORR, I^2^ = 0%; DCR, I^2^ = 30.9%). Compared with targeted therapy alone, oral herbal medicines combined with targeted therapy significantly increased the ORR (RR, 1.49 [1.33-1.66], *P* < 0.0001) and DCR (RR, 1.32 [1.25,1.40], *P* < 0.0001).

#### 3.3.2 Quality of life

**Figure 5:**
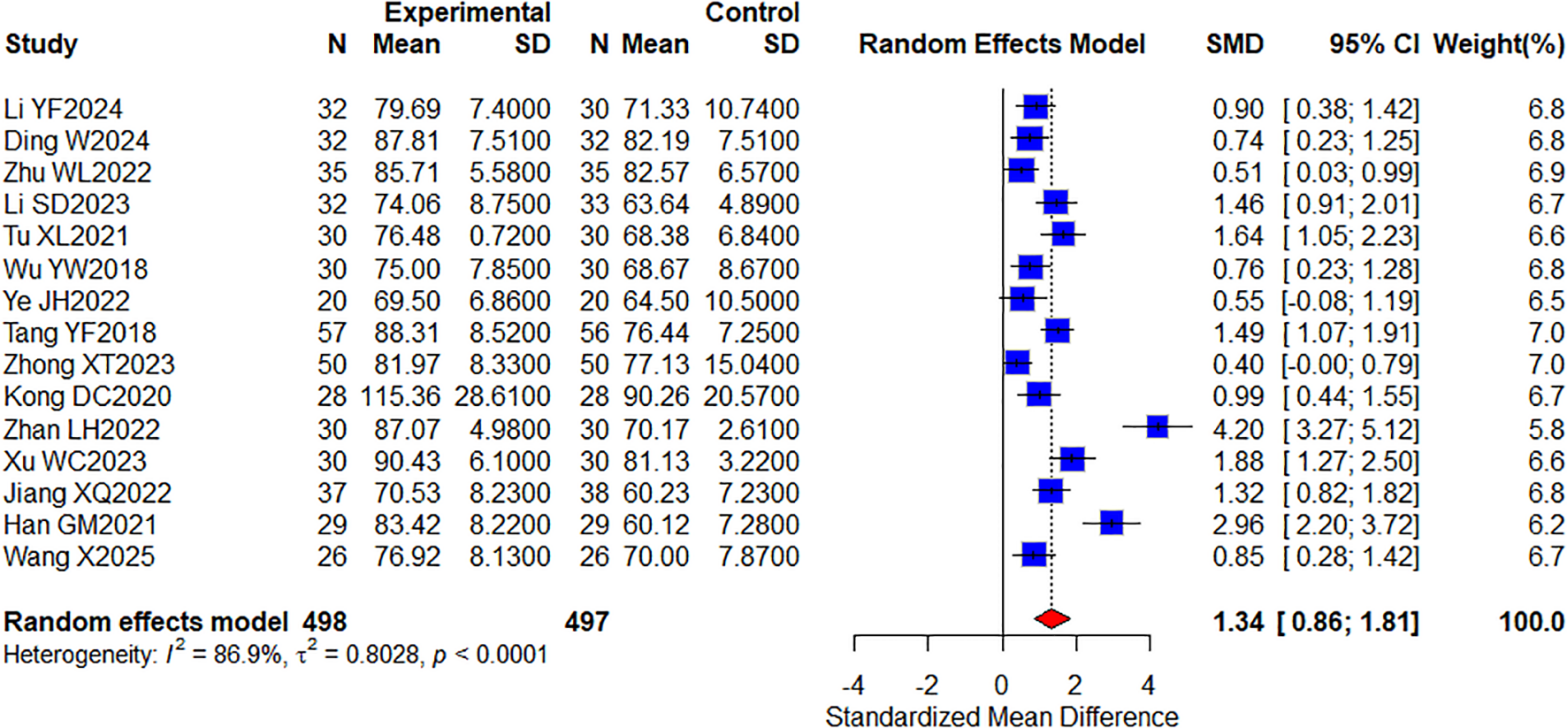
Forest plot of quality of life meta-analysis results.

Among the included studies, a total of 15 studies (29, 32, 42, 43, 45, 46, 48, 55, 63, 64, 66–69, 71) reporting KPS quality of life included 995 cases (**Figure 5**). The results showed that combined TCM significantly improved KPS quality of life compared with targeted therapy alone (SMD, 1.34 [0.86, 1.81]; *P* < 0.0001, I^2^ = 86.9%). In conclusion, KPS was significantly improved in the oral herbal medicine combined with targeted therapy group compared with the control group. In addition, we performed a subgroup analysis of this, which would group the intervention groups according to whether they were combined with other treatment modalities: one group included only targeted therapies; the other group added immune checkpoint inhibitors, and no differences were observed in this result (**Supplementary Figure 1**).

#### 3.3.3 Survival rate

**Figure 6:**
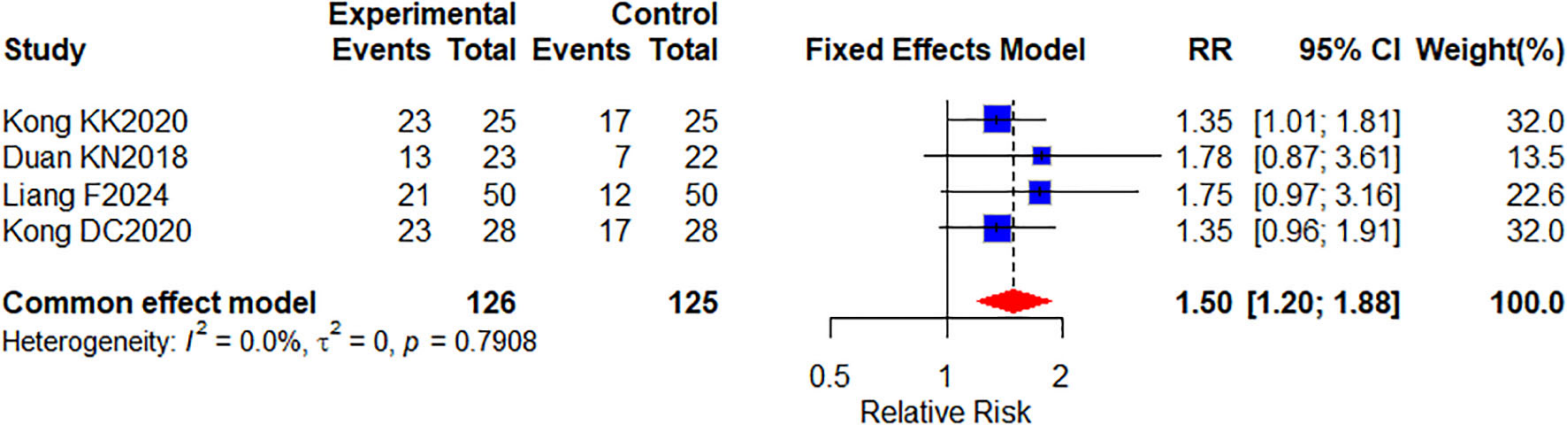
Forest plot of 1-year survival meta-analysis results.

4 studies (26, 34, 62, 64) involving 251 patients reported 1-year survival rates (**Figure 6**).Meta-analysis showed a significant treatment group difference(RR, 1.50 [1.20–1.88], *P* = 0.0004; I^2^ = 0%). These results demonstrate that the combination of oral herbal medicines with targeted therapy plays a significant role in improving 1-year survival rates for CRC patients compared to targeted therapy alone.

#### 3.3.4 Tumor markers

**Figure 7:**
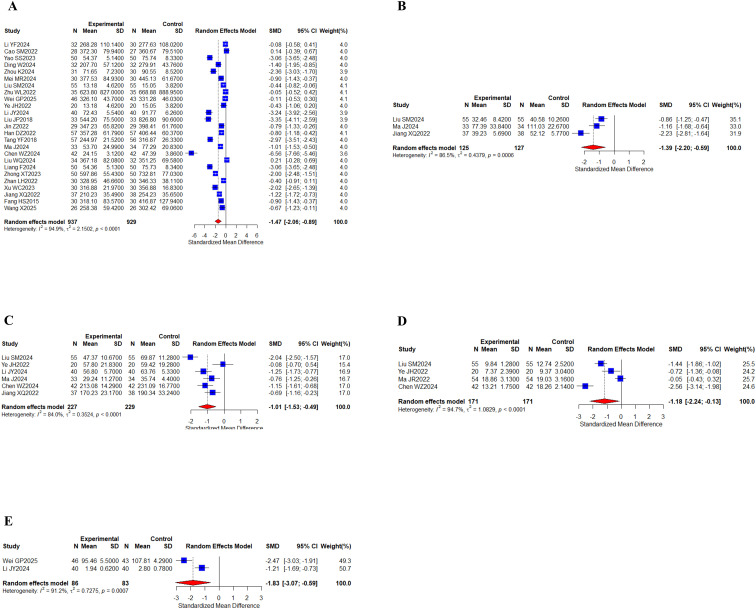
Tumor markers. **(A)** Forest of AFP meta-analysis results; **(B)** Forest of CA125 meta-analysis results; **(C)** Forest of CA199 meta-analysis results; **(D)** Forest of CEA meta-analysis results; **(E)** Forest of CYFRA21–1 meta-analysis results.

A comprehensive analysis of 25 studies (29–32, 38, 40–42, 44, 48, 51–55, 58, 60–63, 66–68, 70, 71) involving 1,866 patients evaluated the AFP biomarker. Additionally, 3 separate studies (41, 58, 68) encompassing 252 participants measured CA125 levels, while 6 research projects (41, 48, 51, 58, 60, 68) with 456 subjects assessed the CA199 marker. 4 clinical investigations (41, 48, 57, 60) totaling 342 patients examined CEA levels, and 2 studies (44, 51) comprising 169 cases reported findings on the CYFRA21–1 biomarker (**Figures 7A–E**). I^2^ values (I^2^ > 80%) indicated high levels of heterogeneity. The results of the study showed that AFP (SMD, -1.47 [-2.05;-0.89], *P* < 0.0001), CA125 (SMD, -1.39 [-2.19;-0.59], *P* = 0.0007), and CA199 (SMD, -1.01 [-1.53;-0.49], *P* = 0.0001) were found between the two groups, CYFRA21-1 (SMD, -1.83 [-3.07;-0.59], *P* = 0.0037) showed statistically significant differences. The statistical results indicated that the group of oral herbal medicines combined with targeted therapy effectively lowered tumor marker levels. Our subgroup analyses, grouped according to whether other treatments were added to the intervention group, showed significant differences in subgroup analyses for the CA199 metrics, with the addition of an immune checkpoint inhibitor to targeted therapy in the intervention group (n = 234, I^2^ = 92%) and targeted therapy only in the intervention group (n = 142, I^2^ = 0%), whereas no differences were observed in the other metrics (**Supplementary Figures 2**-**5**).

#### 3.3.5 Liver function

**Figure 8:**
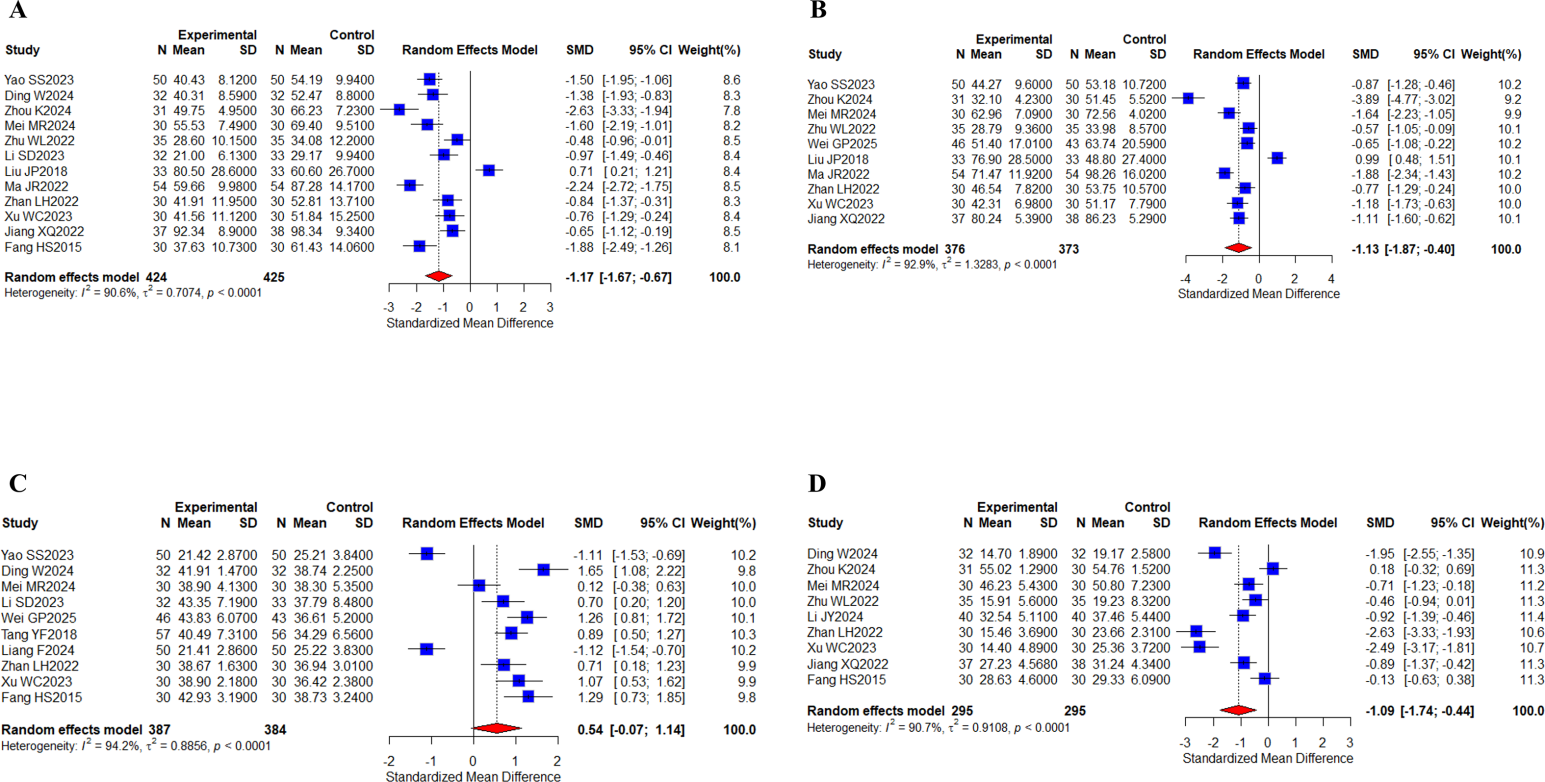
Liver function. **(A)** Forest plot of ALT meta-analysis results; **(B)** Forest plot of AST meta-analysis results; **(C)** Forest plot of ALB meta-analysis results; **(D)** Forest plot of TBIL meta-analysis results.

12 studies (31, 32, 38, 40, 42, 43, 52, 57, 66–68, 70) with a total of 849 cases summarized ALT results; 10 studies (31, 38, 40, 42, 44, 52, 57, 66–68) with a total of 749 cases reported AST results; 10 studies (31, 32, 40, 43, 44, 55, 62, 66, 67, 70) with a total of 771 cases reported ALB indices; and 9 studies (32, 38, 40, 42, 51, 66–68, 70) with a total of 590 cases reported TBIL indices (**Figures 8A–D**). Meta-analysis results showed that I^2^ values (I^2^ > 80%) indicated high levels of heterogeneity. There were statistically significant differences between the results of two groups for ALT (SMD, -1.17 [-1.67;-0.67], *P* < 0.0001), AST (SMD, -1.13 [-1.87;-0.40], *P* = 0.0025), and TBIL (SMD, -1.09 [-1.74;-0.44], *P* = 0.0010), and no statistically significant difference was found in ALB (SMD, 0.54 [-0.07;1.14], *P* = 0.0814) levels. Taken together, the results suggest that the oral herbal medicines combined with a targeted therapy regimen played a significant role in improving liver function, ALT, AST, and TBIL. Our subgroup analyses remained grouped according to the above criteria, and no differences were observed in the results of liver function indices (**Supplementary Figures 6**-**9**).

#### 3.3.6 Peripheral blood lymphocytes

**Figure 9:**
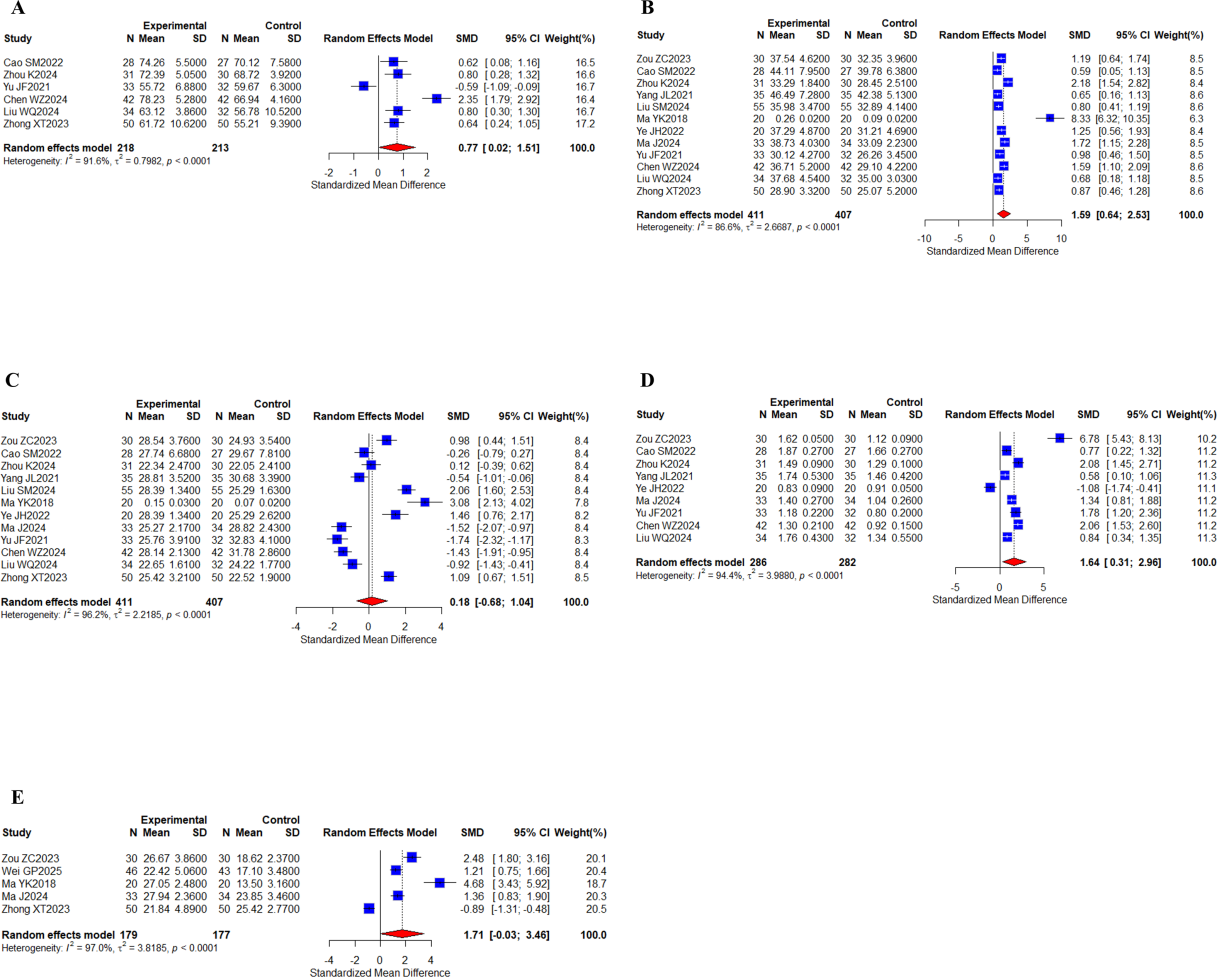
Peripheral blood lymphocytes. **(A)** Forest plot of CD3^+^ meta-analysis results; **(B)** Forest plot of CD4^+^ meta-analysis results; **(C)** Forest plot of DC8^+^ meta-analysis results; **(D)** Forest plot of CD4^+^/CD8^+^ meta-analysis results; **(E)** Forest plot of NK cell meta-analysis results.

6 studies (30, 38, 59–61, 63) with a total of 431 patients tested for CD3+ indicators; 12 studies (23, 30, 38, 39, 41, 47, 48, 58–61, 63) with a total of 818 patients reported CD4^+^, CD8^+^ indicators; 9 studies (23, 30, 38, 39, 48, 58–61) with a total of 568 patients reported CD4^+^/CD8^+^ indicators; and 5 studies (23, 44, 47, 58, 63) with a total of 356 cases reported NK indicators (**Figures 9A–E**). Meta-analysis results showed that I^2^ values (I^2^ > 80%) indicated high levels of heterogeneity. There were statistically significant differences for CD3^+^ (SMD, 0.77 [0.02;1.51], *P* = 0.0439), CD4^+^ (SMD, 1.56 [0.64;2.53], *P* = 0.0010), and CD4^+^/CD8^+^ (SMD, 1.64 [0.31;2.96], *P* = 0.0155), while there was no statistically significant difference in the levels of CD8^+^ (SMD, 0.18 [-0.68;1.04], *P* = 0.6789), NK (SMD, 1.71 [-0.03;3.46], *P* = 0.0539). Taken together, the results suggest that the oral herbal medicines combined with a targeted therapy regimen improved CD3^+^, CD4^+^, and CD4^+^/CD8^+^ levels. We performed subgroup analyses again, and no differences were found in the results (**Supplementary Figures 10**-**14**).

#### 3.3.7 Inflammatory response-related expression

**Figure 10:**
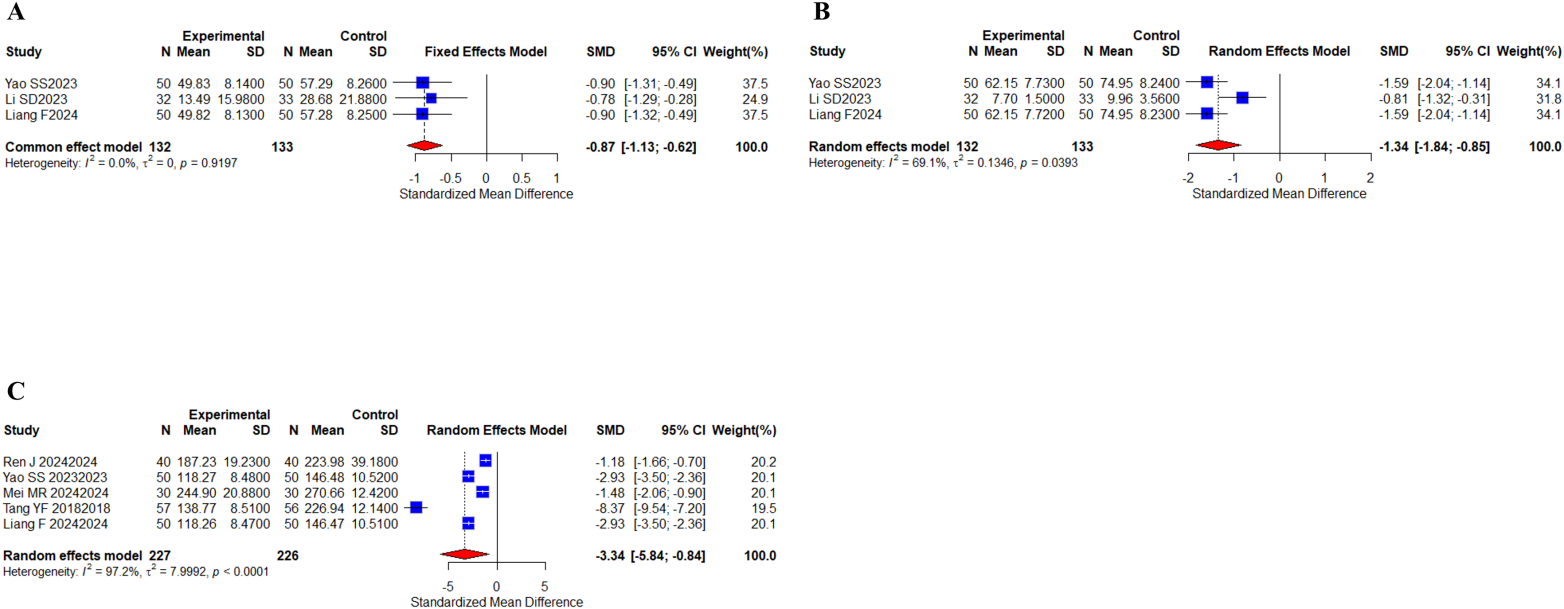
Inflammatory factors. **(A)** Forest plot of IL6 meta-analysis results; **(B)** Forest plot of TNF-α meta-analysis results; **(C)** Forest plot of VEGF meta-analysis results.

3 studies (31, 43, 62) with a total of 265 cases reported IL6 and TNF-alpha metrics; 5 studies (24, 31, 40, 55, 62) with a total of 453 cases reported VEGF metrics (**Figures 10A–C**). The results indicate that the I^2^ value of IL6 metrics (I^2^ = 0%) indicated no heterogeneity; there was a statistically significant difference (SMD, -0.87 [-1.13;- 0.62], *P* < 0.0001). The remaining TNF-α and VEGF index I^2^ values (I^2^ > 60%) indicated a low degree of heterogeneity, which was analyzed using a random-effects model. TNF-α (SMD, -1.34 [-1.84;-0.85], *P* < 0.0001) and VEGF (SMD, -3.34 [-5.84;-0.84], *P* = 0.0087) showed statistically significant differences. Taken together, the results indicated that the oral herbal medicines combined with a targeted therapy regimen improved IL6, TNF-α, and VEGF levels.

#### 3.3.8 Adverse drug reactions

**Figure 11:**
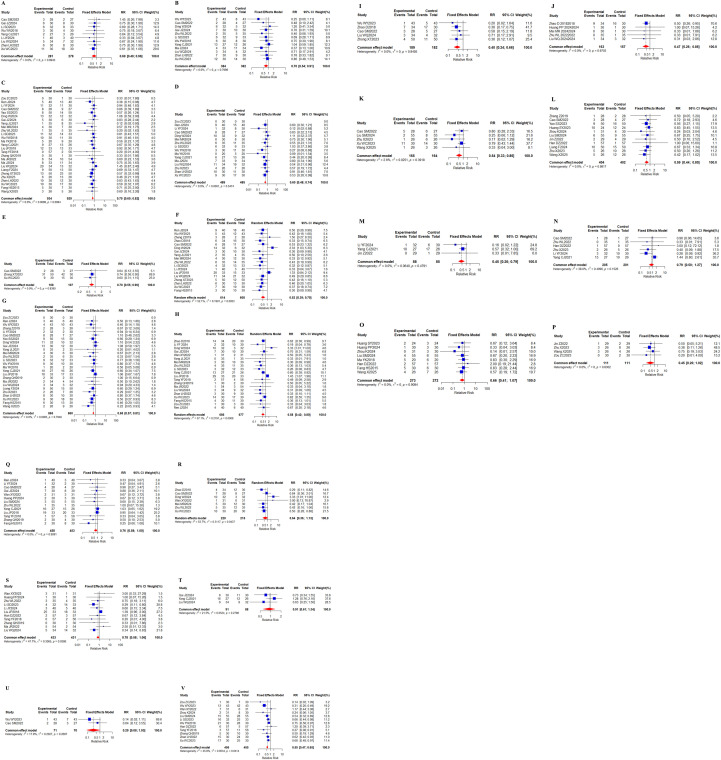
Adverse effects. **(A)** Forest plot of meta-analysis results for leukopenia; **(B)** Forest plot of meta-analysis results for thrombocytopenia; **(C)** Forest plot of meta-analysis results for hypertension; **(D)** Forest plot of meta-analysis results for proteinuria; **(E)** Reactive cutaneous capillary endothelial proliferation meta-analysis result forest map; **(F)** Weakness meta-analysis result forest map; **(G)** Forest plot of meta-analysis results for hand-foot syndrome; **(H)** Forest plot of meta-analysis results for diarrhea; **(I)** Forest plot of results of meta-analysis of hepatic insufficiency; **(J)** Forest plot of results of meta-analysis of renal insufficiency; **(K)** Forest plot of meta-analytic results for thyroid dysfunction; **(L)** Forest plot of meta-analytic results for gastrointestinal reaction; **(M)** Forest plot of meta-analytic results for mouth ulcers; **(N)** Forest plot of meta-analytic results for bleeding; **(O)** Forest plot of meta-analytic results for myelosuppression; **(P)** Forest plot of meta-analytic results for joint/muscle soreness; **(Q)** Forest plot of meta-analytic results for skin rashes; **(R)** Forest plot of meta-analytic results for loss of appetite; **(S)** Forest plot of meta-analysis results for nausea/vomiting; **(T)** Forest plot of meta-analysis results for hemoglobin reduction; **(U)** Forest plot of meta-analytic results for granulocytopenia; **(V)** Forest plot of meta-analytic results for total incidence of adverse events.

38 studies (23–25, 27–33, 35–43, 46, 47, 50–58, 61–63, 65–67, 70, 71) reported the incidence of adverse reactions, specifically including 21 adverse events (**Figures 11A–V**). 18 adverse events showed no heterogeneity (I^2^ <50%). Significant heterogeneity existed for weakness (I^2^ = 52.1%), diarrhea (I^2^ = 57.1%), and loss of appetite (I^2^ = 53.7%). Specific meta-results showed that the overall incidence of adverse effects (RR, 0.55 [0.47,0.65], *P* < 0.0001), leukopenia (RR, 0.69 [0.49,0.96], *P* = 0.0298), and thrombocytopenia (RR, 0.70 [0.54,0.91], *P* = 0.0086) occurred in combination with Chinese herbal medicine and targeted therapy, hypertension (RR, 0.70 [0.60, 0.82], *P* < 0.0001), proteinuria (RR, 0.60 [0.48,0.74], *P* < 0.0001), reactive cutaneous capillary endothelial proliferation (RR, 0.70 [0.55,0.90], *P* = 0.0047), weakness (RR, 0.52 [0.39,0.70], *P* < 0.0001), hand-foot syndrome (RR, 0.68 [0.57,0.81], *P* < 0.0001), diarrhea (RR, 0.58 [0.42,0.80], *P* = 0.0009), hepatic insufficiency (RR, 0.40 [0.24,0.66], *P* = 0.0003), renal insufficiency (RR, 0.47 [0.26 0.86], *P* = 0.0142), thyroid dysfunction(RR, 0.54 [0.33, 0.86], *P* = 0.0101), gastrointestinal reaction (RR, 0.59 [0.44, 0.80], *P* = 0.0006), and mouth ulcers (RR, 0.45 [0.26, 0.79], *P* = 0.0050) incidence rates showed statistically significant difference. In addition, the findings showed in the incidence of bleeding (RR, 0.79 [0.50,1.27], *P* = 0.3297), myelosuppression (RR, 0.66 [0.41, 1.07], *P* = 0.0906), joint/muscle soreness (RR, 0.45 [0.20, 1.02], *P* = 0.0557), and skin rashes (RR, 0.76 [0.58 1.00], *P* = 0.0482), loss of appetite (RR, 0.64 [0.36, 1.13], *P* = 0.1201), nausea/vomiting (RR, 0.78 [0.58, 1.06], *P* = 0.1116), hemoglobin reduction (RR, 0.91 [0.61, 1.34], *P* = 0.6302), and granulocytopenia (RR, 0.29 [0.08, 1.03], *P* = 0.0566) were not statistically significantly different in incidence. Separate subgroup analyses were performed in the indicators of weakness and loss of appetite, according to the addition of immune checkpoint inhibitors to the basis of targeted therapy in the intervention group (n = 215, I^2^ = 0%), (n = 115, I^2^ = 0%), and targeted therapy only in the intervention group (n = 771, I^2^ = 65.8%), (n = 320, I^2^ = 66.6%), whereas no differences were observed in the indicators of diarrhea (**Supplementary Figures 15**-**17**).

#### 3.3.9 TCM syndrome evaluation

**Figure 12:**
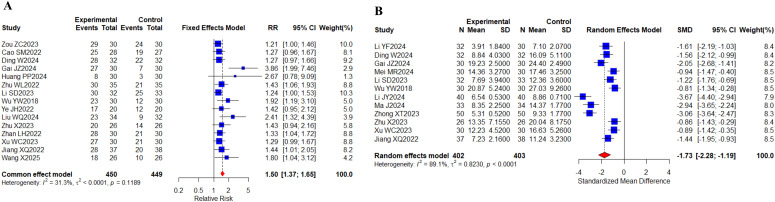
TCM syndrome scores. **(A)** Forest plot of meta-analysis for TCM syndrome composite scores; **(B)** Forest plot of meta-analysis for TCM syndrome efficacy scores.

15 (23, 30, 32, 33, 37, 42, 43, 46, 48, 61, 65–68, 71) studies with a total of 899 cases reported the total TCM syndrome composite scores; 12 (29, 32, 33, 40, 43, 46, 51, 58, 63, 65, 67, 68) studies with a total of 805 cases reported the TCM syndrome efficacy scores (**Figures 12A, B**). Meta results showed that there was no heterogeneity in the total TCM evidence score, I^2^ = 31.3%, and there was significant heterogeneity in the TCM evidence efficacy score, I^2^ = 89.1%. Compared with targeted therapy alone, there were statistically significant differences in the total TCM evidence score (RR, 1.50 [1.37,1.65], *P* < 0.0001) and TCM evidence efficacy score (SMD, -1.73 [-2.28,-1.19], *P* < 0.0001) in the combined TCM group. The above indicates that the combination of oral herbal medicines and targeted therapy plays a good role in improving the total TCM evidence score and TCM evidence efficacy score. No differences were observed in the subgroup analyses (**Supplementary Figure 18**).

### 3.4 Publication bias analysis

**Figure 13:**
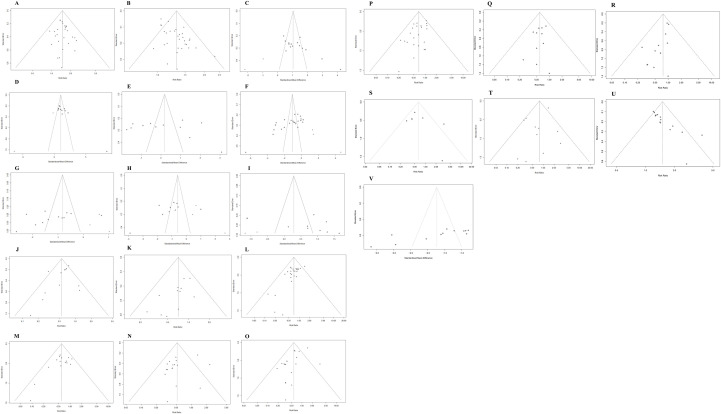
Publication bias evaluation. **(A)** ORR; **(B)** DCR; **(C)** KPS; **(D)** CD4+; **(E)** CD8+; **(F)** AFP; **(G)** ALT; **(H)** AST; **(I)** ALB; **(J)** Total adverse events; **(K)** Thrombocytopenia; **(L)** Hypertension; **(M)** Proteinuria; **(N)** Weakness; **(O)** Diarrhea; **(P)** Hand-foot syndrome; **(Q)** Gastrointestinal reaction; **(R)** Skin rashes; **(S)** Loss of appetite; **(T)** Nausea/vomiting; **(U)** TCM syndrome composite scores; **(V)** TCM syndrome efficacy scores.

We evaluated potential publication bias in our meta-analysis using funnel plots, which were generated for outcomes reported in ≥10 studies. In our analysis, funnel plot examinations were performed for the following parameters: Tumor response: ORR, DCR; Safety profiles: overall adverse events, proteinuria, weakness, nausea/vomiting, diarrhea, hypertension, skin rashes, loss of appetite, reactive cutaneous capillary endothelial proliferation, gastrointestinal reaction, thrombocytopenia; Laboratory markers: AFP, ALB, ALT, AST; Immunological indicators: CD4^+^, CD8^+^; Clinical assessments: KPS; TCM outcomes: TCM syndrome composite scores, TCM syndrome efficacy scores (**Figures 13A–V**). Larger sample sizes enhance result reliability by reducing data variability and standard errors, leading to denser clustering of data points at the funnel plot apex. Smaller samples increase variability and standard errors, resulting in wider dispersion of points at the funnel plot base. As can be seen from the distribution of data points in the figure, most of the studies used larger sample sizes. However, we still included some small-sample studies, all of which provided a thorough description of the intervention and final results. The funnel plot analysis in our research demonstrated approximate symmetry, suggesting no significant publication bias was present.

### 3.5 Sensitivity analysis

Sensitivity analysis determines the impact of key studies on the overall effect size by changing study selection criteria or data assumptions and assessing whether the analysis is robust after recombining the results. To evaluate the reliability of our conclusions, we performed a sensitivity check by sequentially eliminating each study and examining its impact on the combined outcomes. This method helped us identify whether any particular study skewed the meta-analysis findings. When the effect sizes from these sensitivity tests aligned with the primary results, it reinforced the credibility of our overall analysis (**Supplementary Figure 19A**-**U**).

## 4 Discussion

PLC remains a pressing global health concern, marked by rising incidence and mortality rates. While molecular targeted therapies (*e.g*., sorafenib, lenvatinib, regorafenib) have revolutionized treatment, their clinical utility is constrained by drug resistance, adverse events, and high costs (72). TCM has become an important adjuvant therapy in tumor treatment and plays an active role in the clinic (73). While TCM adopts a holistic approach to treating PLC, modern research has uncovered novel anti-tumor mechanisms of its active compounds. These findings provide a mechanistic rationale for the synergistic effects of TCM and represent a crucial step in its modernization. Accumulating evidence indicates that the anti-tumor mechanisms of TCM are multifaceted, including inducing apoptosis and autophagy, promoting cell cycle arrest, inhibiting angiogenesis and metastasis, as well as exerting anti-inflammatory, antioxidant, and immunomodulatory effects (16). The active components of the Chinese herbal formula Wan-Nian-Qing inhibit the growth of PLC cells by modulating the expression of serum interleukins, chemokines, and tumor necrosis factor. This immunomodulation, in turn, activates natural killer (NK) cells and regulates T-cell responses, ultimately leading to the promotion of tumor cell apoptosis (74). It is precisely due to properties such as anti-inflammatory, immunomodulatory, and anti-angiogenic effects that TCM supports its application across all stages of PLC development and progression. Numerous studies have reported that TCM combined with targeted-based therapeutic regimens can provide survival benefits to patients with advanced PLC. However, their effectiveness and safety have not been systematically and comprehensively analyzed. Therefore, this study systematically analyzed and included 49 randomized controlled clinical studies to evaluate the efficacy and safety of targeted-based regimens combined with oral herbal medicine in the treatment of advanced PLC. The analysis revealed significantly higher ORR and DCR in the treatment group compared to targeted therapy alone. Therefore, the targeted-based regimen proved to be more effective when combined with oral herbal medicine.

Growing evidence underscores the immune function’s key role in tumor development and advancement, making immune status and quality-of-life assessments essential for cancer patients undergoing pharmacotherapy (75). CD3, a characteristic surface marker of T cells, constitutes a complex that plays a key role in antigen recognition and signaling (76). Furthermore, CD4^+^ T cells, functioning as helper T cell subsets, serve as central regulators of antitumor immune responses through cytokine secretion and CD8^+^ T cell activation, representing key modulators of adaptive immunity. Astragaloside enhances the secretion of interleukin-2 (IL-2) and interferon-gamma (IFN-γ), thereby promoting the expression of CD25 and CD69 on CD4+ T cells (77). In contrast, CD8^+^ cytotoxic T lymphocytes (CTLs) directly eliminate tumor cells via effector molecules, including granzymes. In clinical practice, the CD4^+^/CD8^+^ ratio serves as a critical biomarker for evaluating immune homeostasis (78). Quantification of these surface markers has become an essential clinical tool for assessing immune function, monitoring tumor progression, and predicting therapeutic outcomes and prognosis (79). TCM exhibits multi-pathway and multi-target mechanisms of action with favorable safety profiles, demonstrating efficacy in modulating the tumor immune microenvironment and enhancing therapeutic outcomes (20, 80). Various herbal decoctions have gained widespread clinical application due to their convenient administration and demonstrated treatment benefits. Therefore, this study conducted a meta-analysis to assess the effectiveness and safety of integrating targeted therapy and oral TCM in PLC treatment. Our findings demonstrate that the combined regimen significantly improved CD3^+^ and CD4^+^ T cell counts and CD4^+^/CD8^+^ ratio and reduced the expression of inflammation-associated factors compared to targeted therapy alone. In addition, it has largely improved the overall quality of life of patients with advanced PLC. Research indicates (81) that herbs regulate immunity by reducing inflammation and boosting immune activity. *In vivo* experiments showed (82) that rhubarb stinging pill could reverse the Treg/TH1 balance of CD4^+^ T cells and improve the suppressed immune state of the body. These findings demonstrate that herbal medicine exerts antitumor effects by modulating T-cell immunity and suppressing inflammation. Our results suggest that distinct Chinese herbal formulations can remodel the tumor microenvironment and enhance immune function.

Tumor biomarkers, as bioactive substances produced by malignant cells or their microenvironment, play pivotal roles in early detection, therapeutic monitoring, and prognosis assessment of cancers (83). AFP remains the gold-standard diagnostic marker for PLC (84), with elevated levels constituting both a risk factor (85) and a reliable indicator for diagnosis, prognosis, and surveillance (86). CA125 elevation correlates with aggressive tumor biology and poor outcomes in PLC (87). CA199, while more characteristic of ICC, retains prognostic value in PLC (88). Our study quantitatively evaluated serum levels of key tumor biomarkers (AFP, CA125, CA199), revealing that the oral herbal medicine combined with target-based regimens also has the potential to reduce the expression levels of relevant tumor markers, which can significantly improve the therapeutic efficacy.

Finally, the results suggest that a targeted regimen based on the combined use of oral herbal medicines may reduce the overall incidence of adverse events. The study revealed that patients receiving combination therapy experienced fewer adverse effects, including leukopenia, thrombocytopenia, hypertension, proteinuria, reactive cutaneous capillary endothelial proliferation, weakness, hand-foot syndrome, diarrhea, hepatic/renal insufficiency, thyroid dysfunction, gastrointestinal reaction, and mouth ulcers as compared to the control group. Therefore, the results obtained from the analysis of this study support that the combination therapy based on a targeted therapeutic regimen with oral herbal medicine is safe for the treatment of PLC. In addition, liver function indexes are also commonly used to assess drug safety and are crucial in PLC treatment and prognosis (89). The results showed that the combination therapy group significantly improved the ALT, AST, and TBIL levels of patients, thus improving liver function. It has been shown (90) that tiliroside increases the activity of sorafenib in hepatocellular carcinoma by inducing iron death, and no side effects have been observed as a result.

The enhanced tumor response rate observed in this meta-analysis is particularly noteworthy. This challenges the traditional view of TCM as a purely supportive therapeutic modality. We hypothesize that this synergistic effect may stem from TCM’s multi-targeted mechanisms of action, such as reversing multidrug resistance by inhibiting efflux pumps, suppressing pro-angiogenic factors beyond VEGF, and modulating the tumor microenvironment (91). Furthermore, the significant reduction in both the incidence and severity of adverse events provides direct and immediate guidance for clinical practice. Managing side effects is crucial for maintaining the dose intensity of targeted therapy—a factor proven to predict survival outcomes (92). By mitigating toxic reactions, TCM indirectly prolongs the duration patients can receive effective treatment, thereby promoting survival benefits. For clinicians, this study provides preliminary evidence supporting the integration of standardized TCM interventions, particularly for patients burdened by treatment-related symptoms. This strategy aligns with precision supportive care objectives, aiming for personalized management focused not only on the tumor but on the patient as a whole.

This review has several limitations, while also pointing to clear directions for future research. First, variations exist in TCM interventions, such as different herbal formulas and treatment cycles. Future studies should shift from exploring “whether TCM is effective” to investigating “which herbal formula is most effective for specific patient characteristics” or “when is the optimal timing for integrated treatment.” Second, the number of included trials is limited, with insufficient data for certain indicators. Most studies originated from single-center investigations conducted in China, raising potential concerns about regional bias that may lead to heterogeneity in results. Therefore, we recommend that future studies incorporate multicenter, large-sample, high-quality randomized controlled trials to support our findings and enhance the robustness of research.

## 5 Conclusions

In summary, this meta-analysis provides compelling evidence that the combination of TCM with targeted therapy offers a synergistic strategy to enhance treatment efficacy and reduce toxicity in PLC. These findings hold significant clinical implications, suggesting that TCM should be considered a valuable therapeutic modality within the modern oncology treatment framework. However, translating these findings into routine clinical practice requires prioritizing the standardization of TCM interventions in future research. This necessitates conducting multicenter, large-scale, high-quality, rigorously controlled trials that incorporate mechanistic studies. Such efforts are essential for validating current results, defining specific clinical application, and ultimately paving the way for integrating TCM into PLC treatment protocols.

## Data Availability

The original contributions presented in the study are included in the article/**Supplementary Material**. Further inquiries can be directed to the corresponding authors.

## References

[1] Bray F Laversanne M Sung H Ferlay J Siegel RL Soerjomataram I . Global cancer statistics 2022: GLOBOCAN estimates of incidence and mortality worldwide for 36 cancers in 185 countries. CA Cancer J Clin. (2024) 74:229–63. doi: 10.3322/caac.21834, PMID: 38572751

[2] Wang Y Deng B . Hepatocellular carcinoma: molecular mechanism, targeted therapy, and biomarkers. Cancer Metastasis Rev. (2023) 42:629–52. doi: 10.1007/s10555-023-10084-4, PMID: 36729264

[3] Kim H Jang M Kim E . Exploring the multifunctional role of alpha-fetoprotein in cancer progression: implications for targeted therapy in hepatocellular carcinoma and beyond. Int J Mol Sci. (2025) 26:4863. doi: 10.3390/ijms26104863, PMID: 40430002 PMC12112184

[4] Hu X Chen R Wei Q Xu X . The landscape of alpha fetoprotein in hepatocellular carcinoma: where are we? Int J Biol Sci. (2022) 18:536–51. doi: 10.7150/ijbs.64537, PMID: 35002508 PMC8741863

[5] Liu W Cao S Shi D Yu L Qiu W Chen W . Single-chemical and mixture effects of multiple volatile organic compounds exposure on liver injury and risk of non-alcoholic fatty liver disease in a representative general adult population. Chemosphere. (2023) 339:139753. doi: 10.1016/j.chemosphere.2023.139753, PMID: 37553041

[6] Park JW Chen M Colombo M Roberts LR Schwartz M Chen PJ . Global patterns of hepatocellular carcinoma management from diagnosis to death: the BRIDGE Study. Liver Int. (2015) 35:2155–66. doi: 10.1111/liv.12818, PMID: 25752327 PMC4691343

[7] Altekruse SF McGlynn KA Reichman ME . Hepatocellular carcinoma incidence, mortality, and survival trends in the United States from 1975 to 2005. J Clin Oncol. (2009) 27:1485–91. doi: 10.1200/JCO.2008.20.7753, PMID: 19224838 PMC2668555

[8] Singal AG Yarchoan M Yopp A Sapisochin G Pinato DJ Pillai A . Neoadjuvant and adjuvant systemic therapy in HCC: Current status and the future. Hepatol Commun. (2024) 8:e0430. doi: 10.1097/HC9.0000000000000430, PMID: 38829199 PMC11150030

[9] Yang C Zhang H Zhang L Zhu AX Bernards R Qin W . Evolving therapeutic landscape of advanced hepatocellular carcinoma. Nat Rev Gastroenterol Hepatol. (2023) 20:203–22. doi: 10.1038/s41575-022-00704-9, PMID: 36369487

[10] Reig M Forner A Rimola J Ferrer-Fàbrega J Burrel M Garcia-Criado Á . BCLC strategy for prognosis prediction and treatment recommendation: The 2022 update. J Hepatol. (2022) 76:681–93. doi: 10.1016/j.jhep.2021.11.018, PMID: 34801630 PMC8866082

[11] Llovet JM Pinyol R Kelley RK El-Khoueiry A Reeves HL Wang XW . Molecular pathogenesis and systemic therapies for hepatocellular carcinoma. Nat Cancer. (2022) 3:386–401. doi: 10.1038/s43018-022-00357-2, PMID: 35484418 PMC9060366

[12] Jiao Q Bi L Ren Y Song S Wang Q Wang YS . Advances in studies of tyrosine kinase inhibitors and their acquired resistance. Mol Cancer. (2018) 17:36. doi: 10.1186/s12943-018-0801-5, PMID: 29455664 PMC5817861

[13] Kudo M Finn RS Qin S Han KH Ikeda K Piscaglia F . Lenvatinib versus sorafenib in first-line treatment of patients with unresectable hepatocellular carcinoma: a randomised phase 3 non-inferiority trial. Lancet. (2018) 391:1163–73. doi: 10.1016/S0140-6736(18)30207-1, PMID: 29433850

[14] Abou-Alfa GK Lau G Kudo M Chan SL Kelley RK Furuse J . Tremelimumab plus durvalumab in unresectable hepatocellular carcinoma. NEJM Evid. (2022) 1:EVIDoa2100070. doi: 10.1056/EVIDoa2100070, PMID: 38319892

[15] Cheng AL Qin S Ikeda M Galle PR Ducreux M Kim TY . Updated efficacy and safety data from IMbrave150: Atezolizumab plus bevacizumab vs. sorafenib for unresectable hepatocellular carcinoma. J Hepatol. (2022) 76:862–73. doi: 10.1016/j.jhep.2021.11.030, PMID: 34902530

[16] Xu AX Zhao ZF Zhu L Zhang YH Li Y Wei YF . Promise and challenges of traditional Chinese medicine, specifically Calculus bovis, in liver cancer treatment. World J Gastroenterol. (2024) 30:4380–5. doi: 10.3748/wjg.v30.i40.4380, PMID: 39494098 PMC11525868

[17] Li YC Zhang HX . Overview of mechanism of TCM prevention and treatment of liver cancer. J Pract Tradit Chin Intern Med. (2024) 38:43–7. doi: 10.13729/j.issn.1671-7813.Z20231723

[18] Wang S Long S Deng Z Wu W . Positive role of chinese herbal medicine in cancer immune regulation. Am J Chin Med. (2020) 48:1577–92. doi: 10.1142/S0192415X20500780, PMID: 33202152

[19] Liu X Li M Wang X Dang Z Yu L Wang X . Effects of adjuvant traditional Chinese medicine therapy on long-term survival in patients with hepatocellular carcinoma. Phytomedicine. (2019) 62:152930. doi: 10.1016/j.phymed.2019.152930, PMID: 31128485

[20] Page MJ McKenzie JE Bossuyt PM Boutron I Hoffmann TC Mulrow CD . The PRISMA 2020 statement: an updated guideline for reporting systematic reviews. BMJ. (2021) 372:n71. doi: 10.1136/bmj.n71, PMID: 33782057 PMC8005924

[21] Miller AB Hoogstraten B Staquet M Winkler A . Reporting results of cancer treatment. Cancer. (1981) 47:207–14. doi: 10.1002/1097-0142(19810101)47:1207::aid-cncr28204701343.0.co;2-6 7459811

[22] Eisenhauer EA Therasse P Bogaerts J Schwartz LH Sargent D Ford R . New response evaluation criteria in solid tumours: revised RECIST guideline (version 1.1). Eur J Cancer. (2009) 45:228–47. doi: 10.1016/j.ejca.2008.10.026, PMID: 19097774

[23] Zou ZC Yang HZ Li YW . Clinical efficacy of Yin-Yang Gongji Pill combined with hepatic TACE and targeted immunotherapy (sintilimab + lenvatinib) in the treatment of intermediate-advanced hepatocellular carcinoma. Inner Mongolia J Traditional Chin Med. (2023) 42:23–4. doi: 10.16040/j.cnki.cn15-1101.2023.11.019

[24] Ren J Liu HJ Shen LL Cao ZJ Zhai XF Wang XL . Clinical study of Yiqi Jiedu Fang in treating primary hepatocellular carcinoma. Henan Traditional Chin Med. (2024) 44:744–8. doi: 10.16367/j.issn.1003-5028.2024.05.0138

[25] Wu WY . Clinical efficacy of Yiqi Huayu Jiedu Decoction combined with Western medicine in treating primary hepatocellular carcinoma. Chin Foreign Med Res. (2023) 2:99–101.

[26] Kong KK . Effect of Yiqi Huayu Jiedu Decoction combined with sorafenib on prognosis of patients with primary hepatocellular carcinoma. Modern Diagn Treat. (2020) 31:1026–8.

[27] Zhang Z Gao WH Wang YQ Li KX Zeng PH . Therapeutic efficacy of modified Yiqi Huayu Jiedu Formula combined with sorafenib in primary hepatocellular carcinoma. Shaanxi J Traditional Chin Med. (2019) 40:322–4. doi: 10.3969/j.issn.1000-7369.2019.03.014

[28] Zhao D Wei HL Li JT Yan SG Guo H Si MM . Yipi Yanggan Formula combined with sorafenib in 34 cases of intermediate-advanced hepatocellular carcinoma. Modern Traditional Chin Med. (2018) 38:31–3. doi: 10.13424/j.cnki.mtcm.2018.01.012

[29] Li YF . Clinical efficacy observation of modified Yiguanjian Decoction combined with lenvatinib in treating intermediate-advanced primary hepatocellular carcinoma with liver-kidney yin deficiency syndrome. Haerbin, HLJ: Heilongjiang University of Chinese Medicine (2024). doi: 10.27127/d.cnki.ghlzu.2024.000458

[30] Cao SM . Clinical efficacy observation of Yangzheng Xiaoji Capsule combined with camrelizumab and apatinib in the treatment of intermediate-advanced hepatocellular carcinoma. Hefei, AH: Anhui University of Chinese Medicine (2023). doi: 10.26922/d.cnki.ganzc.2023.000618

[31] Yao SS Yan TF Wang JL Xu HJ Zhang GL . Clinical observation of Xuanyu Huadu Decoction combined with sorafenib for postoperative recurrent hepatocellular carcinoma. World J Integrated Traditional Western Med. (2023) 18:2081–5. doi: 10.13935/j.cnki.sjzx.231031

[32] Ding W . Clinical efficacy observation of modified Xiaoyao San combined with lenvatinib for primary hepatocellular carcinoma with liver depression-spleen deficiency pattern. Haerbin, HLJ: Heilongjiang University of Chinese Medicine (2024). doi: 10.27127/d.cnki.ghlzu.2024.000423

[33] Gai JZ . Correlation analysis between clinical characteristics and TCM syndromes in advanced primary hepatocellular carcinoma, and clinical study of Shugan Jianpi Huazhuo Formula combined with sorafenib for liver depression-spleen deficiency type advanced HCC. Tianjin, TJ: Tianjin University of Traditional Chinese Medicine (2024). doi: 10.27368/d.cnki.gtzyy.2024.000411

[34] Duan KN . Therapeutic analysis of sorafenib combined with syndrome-differentiated Chinese herbal decoction for advanced primary hepatocellular carcinoma. World Latest Med Inf. (2018) 18:151–2. doi: 10.19613/j.cnki.1671-3141.2018.105.073

[35] Wan XY . Therapeutic effect observation of sorafenib combined with modified Xiaoyao San for primary hepatocellular carcinoma. Contemp Med Symp. (2022) 20:160–2.

[36] Huang SY Zhang Y Zhang SL Zeng XM . Therapeutic efficacy of Siteng Formula combined with modified Yinchenhao Decoction for advanced hepatocellular carcinoma. J Med Theory Pract. (2023) 36:3658–60. doi: 10.19381/j.issn.1001-7585.2023.21.020

[37] Huang PP Shu Y Sun H Chen T Jiang YT Ma MJ . Clinical study of Sini Decoction as adjuvant therapy for 30 cases of advanced primary hepatocellular carcinoma with yang deficiency pattern. Jiangsu J Traditional Chin Med. (2024) 56:39–42. doi: 10.19844/j.cnki.1672-397X.2024.07.011

[38] Zhou K Yuan W Wu J Xia Z Zhang F . Clinical efficacy of Shugan Jianpi Jiedu Formula combined with tislelizumab and bevacizumab for advanced hepatocellular carcinoma. Chin Traditional Patent Med. (2024) 46:4221–4. doi: 10.3969/j.issn.1001-1528.2024.12.052

[39] Yang JL Lin AQ Lu ZN . Clinical efficacy of regorafenib combined with sijunzi decoction in the treatment of primary liver cancer. World Latest Med Inf (Electronic Continuous Journal). (2021) 21:254–5. doi: 10.3969/j.issn.1671-3141.2021.27.108

[40] Mei MR . Clinical Observation of Rougan Sanjie Pills Combined with Lenvatinib in Treating Primary Liver Cancer with Qi Stagnation and Blood Stasis Pattern. Nanning, GX: Guangxi University of Chinese Medicine (2024). doi: 10.27879/d.cnki.ggxzy.2024.000611

[41] Liu SM Chen M . Efficacy and safety of Qinggan Xiaozheng Formula combined with camrelizumab and lenvatinib for elderly patients with hepatocellular carcinoma. Chin J Gerontol. (2024) 44:2841–4. doi: 10.3969/j.issn.1005-9202.2024.12.007

[42] Zhu WL . Effects of Qi'e Baogan Formula combined with targeted drugs on adverse reactions in patients with primary hepatocellular carcinoma. Nanning, GX: Guangxi University of Chinese Medicine (2022). doi: 10.27879/d.cnki.ggxzy.2022.000259

[43] Li SD . Efficacy evaluation of Poyu Jiedu Formula combined with lenvatinib in intermediate-advanced hepatocellular carcinoma and its effects on inflammatory factors TNF-α, IL-6, and IL-17. Zhengzhou, HN: Henan University of Chinese Medicine (2023). doi: 10.27119/d.cnki.ghezc.2023.000462

[44] Wei GP . Clinical study of Peiyuan Guben Formula combined with anlotinib hydrochloride capsules in the treatment of primary hepatocellular carcinoma. Henan Traditional Chin Med. (2025) 45:289–93. doi: 10.16367/j.issn.1003-5028.2025.02.0048

[45] Tu XL Shi GJ Zhang TS Wang YS Hu MY . Clinical observation of Jianpi Yanggan Jiedu Formula combined with lenvatinib in the treatment of advanced primary hepatocellular carcinoma. Chin J Traditional Med Sci Technol. (2021) 28:781–2.

[46] Wu YW . Clinical observation of spleen-strengthening and liver-soothing therapy combined with apatinib in the treatment of intermediate-advanced hepatocellular carcinoma. Guangzhou, GD: Guangzhou University of Chinese Medicine (2018).

[47] Ma YK Fang SM Chen L Yang JZ Yang XY Shen TH . Clinical efficacy of Jianpi Jiedu Formula combined with apatinib mesylate tablets in the treatment of advanced primary hepatocellular carcinoma. Hebei J Traditional Chin Med. (2018) 40:1682–6. doi: 10.3969/j.issn.1002-2619.2018.11.019

[48] Ye JH Fang Z . Clinical observation of Jianpi Huoxue Formula combined with camrelizumab and lenvatinib in the treatment of hepatocellular carcinoma. Shanxi J Traditional Chin Med. (2022) 38:35–7. doi: 10.20002/j.issn.1000-7156.2022.06.012

[49] Sun Y . Effects of modified Yiguanjian Decoction combined with apatinib mesylate on therapeutic efficacy and quality of life in patients with advanced primary hepatocellular carcinoma. J Yunnan Traditional Chin Med Materia Med. (2019) 40:56–7. doi: 10.16254/j.cnki.53-1120/r.2019.04.025

[50] Yang CJ . Clinical observation of modified Xiaochaihu Decoction combined with sorafenib in the treatment of intermediate-advanced primary hepatocellular carcinoma. Chengdu, SC: Chengdu University of Traditional Chinese Medicine (2021). doi: 10.26988/d.cnki.gcdzu.2021.000224

[51] Li JY Li Y Yin X . Therapeutic effects of Jianpi Xingqi Jiedu therapy (spleen-strengthening, qi-moving, and detoxification method) based on the "preventing disease progression" principle in intermediate-advanced primary hepatocellular carcinoma. Henan Med Res. (2024) 33:1487–90. doi: 10.3969/j.issn.1004-437X.2024.08.038

[52] Liu JP Cao JG Yuan CJ Zou XJ Lu W Liu L . Efficacy evaluation of Qinghuo Tongluo Formula combined with sorafenib in hepatocellular carcinoma treatment based on "Xiang thinking" theory. J Hubei Univ Chin Med. (2018) 20:22–5. doi: 10.3969/j.issn.1008-987x.2018.05.05

[53] Jin Z Xie YH He C Wu Q . Clinical observation of Huisheng Oral Liquid combined with lenvatinib in the treatment of advanced hepatocellular carcinoma. Chin Folk Therapies. (2022) 30:120–3. doi: 10.19621/j.cnki.11-3555/r.2022.2432

[54] Han DZ . Therapeutic effects of Huaier Granule combined with sorafenib on postoperative recurrent primary hepatocellular carcinoma and its impacts on AFP and AFP-L3 levels. China J Pharm Econ. (2022) 17:64–7. doi: 10.12010/j.issn.1673-5846.2022.01.013

[55] Tang YF Zhu XJ Huang LY Zhang X Zheng C Gao YQ . Clinical study of Huaier Granule combined with sorafenib in the treatment of advanced hepatocellular carcinoma. Drugs Clinic. (2018) 33:1732–5. doi: 10.7501/j.issn.1674-5515.2018.07.039

[56] Zhang QH Liang YH . Clinical efficacy of Huaier Granule combined with sorafenib in postoperative recurrent primary hepatocellular carcinoma. Pract J Cancer. (2019) 34:1560–2. doi: 10.3969/j.issn.1001-5930.2019.09.049

[57] Ma JR Zhang K . Efficacy of Huazhi Rougan Granules in regorafenib-targeted therapy for intermediate-advanced hepatocellular carcinoma patients with dampness-heat accumulation syndrome. J Clin Res Med. (2022) 39:510–3. doi: 10.3969/j.issn.1671-7171.2022.04.009

[58] Ma J . Clinical study of Compound Shougong Powder combined with lenvatinib in the treatment of advanced hepatocellular carcinoma with qi deficiency and blood stasis syndrome. Hefei, AH: Anhui University of Chinese Medicine (2024). doi: 10.26922/d.cnki.ganzc.2024.000588

[59] Yu JF Li ZP Zhou XL Li C Liu Y Zhang Y . Therapeutic efficacy of Fuling Sini Decoction combined with sorafenib in advanced primary hepatocellular carcinoma. Acta Chin Med Pharmacol. (2021) 49:76–80. doi: 10.19664/j.cnki.1002-2392.210143

[60] Chen WZ Zhang XJ Cai LY . Effects of Fuling Sini Decoction combined with camrelizumab and apatinib on short-term efficacy and lymphocyte subset levels in patients with advanced primary hepatocellular carcinoma. Harbin Med J. (2024) 44:123–5. doi: 10.3969/j.issn.1001-8131.2024.06.041

[61] Liu WQ . Efficacy observation of Fuzheng Sanjie Formula combined with low-dose apatinib in treating advanced hepatocellular carcinoma with spleen qi deficiency and phlegm-heat stasis syndrome. Hefei, AH: Anhui University of Chinese Medicine (2024). doi: 10.26922/d.cnki.ganzc.2024.000693

[62] Liang F Yan TF Wang JL Xu HJ Liang H . Clinical observation of Fuzheng Anzhong Decoction combined with sorafenib in patients with intermediate-advanced hepatocellular carcinoma. World Chin Med. (2024) 19:377–82. doi: 10.3969/j.issn.1673-7202.2024.03.014

[63] Zhong XT . Clinical efficacy observation of Fuhe Beihua Formula combined with camrelizumab and apatinib in treating advanced hepatocellular carcinoma (liver depression-spleen deficiency pattern) after TACE. Nanning, GX: Guangxi University of Chinese Medicine (2023). doi: 10.27879/d.cnki.ggxzy.2023.000369

[64] Kong DC Tian ZR . Analysis of the effect of modified Yiqi Huayu Jiedu Formula combined with sorafenib on survival rate in patients with primary hepatocellular carcinoma. Heilongjiang J Traditional Chin Med. (2020) 49:100–1.

[65] Zhu X . Clinical efficacy observation of modified Danzhi Xiaoyao Powder combined with lenvatinib in treating advanced primary hepatocellular carcinoma with liver depression-spleen deficiency syndrome. Chengde, HB: Chengde Medical University (2023). doi: 10.27691/d.cnki.gcdyx.2023.000178

[66] Zhan LH . Clinical study of Chaishao Decoction combined with apatinib mesylate tablets in the treatment of primary hepatocellular carcinoma (liver depression and spleen deficiency pattern) after TACE. Nanning, GX: Guangxi University of Chinese Medicine (2022). doi: 10.27879/d.cnki.ggxzy.2022.000146

[67] Xu WC . Clinical study of Chaiping Decoction combined with lenvatinib and camrelizumab in the treatment of unresectable hepatocellular carcinoma after TACE. Nanning, GX: Guangxi University of Chinese Medicine (2023). doi: 10.27879/d.cnki.ggxzy.2023.000271

[68] Jiang XQ Wang LN . Clinical efficacy of Chaihu Shugan Huayu Formula combined with lenvatinib in treating primary hepatocellular carcinoma (Qi stagnation and blood stasis syndrome). Chin J Integrated Traditional Western Med Liver Dis. (2022) 32:462–4. doi: 10.3969/j.issn.1005-0264.2022.05.021

[69] Han GM . Clinical observation of Chaihu Biejia Decoction combined with sorafenib in the treatment of advanced hepatocellular carcinoma. Chin Med Modern Distance Educ China. (2021) 19:148–50. doi: 10.3969/j.issn.1672-2779.2021.02.059

[70] Fang HS . Clinical Study and Mechanism Exploration of Shentao Ruangan Formula Combined with Sorafenib in the Treatment of Intermediate-Advanced Primary Liver Cancer. Guangzhou, GD: Guangzhou University of Chinese Medicine (2015).

[71] Wang X Xia LM . Clinical efficacy of Shenqi Xiaoji Formula combined with tislelizumab and lenvatinib in treating intermediate-advanced hepatocellular carcinoma with Zhengxu Yujie syndrome (deficiency-excess stasis pattern). Clin J Traditional Chin Med. (2025) 37:146–52. doi: 10.16448/j.cjtcm.2025.0134

[72] Zheng J Cai J Tao L Kirih MA Shen Z Xu J . Comparison on the efficacy and prognosis of different strategies for intrahepatic recurrent hepatocellular carcinoma: A systematic review and Bayesian network meta-analysis. Int J Surg. (2020) 83:196–204. doi: 10.1016/j.ijsu.2020.09.031, PMID: 32980518

[73] Tang JL Liu BY Ma KW . Traditional chinese medicine. Lancet. (2008) 372:1938–40. doi: 10.1016/S0140-6736(08)61354-9, PMID: 18930523

[74] Zhang X Liu X Zhang Y Yang A Zhang Y Tong Z . Wan-nian-qing, a herbal composite prescription, suppresses the progression of liver cancer in mice by regulating immune response. Front Oncol. (2021) 11:696282. doi: 10.3389/fonc.2021.696282, PMID: 34307161 PMC8297951

[75] Hanna RN Cekic C Sag D Tacke R Thomas GD Nowyhed H . Patrolling monocytes control tumor metastasis to the lung. Science. (2015) 350:985–90. doi: 10.1126/science.aac9407, PMID: 26494174 PMC4869713

[76] Alcover A Alarcón B Di Bartolo V . Cell biology of T cell receptor expression and regulation. Annu Rev Immunol. (2018) 36:103–25. doi: 10.1146/annurev-immunol-042617-053429, PMID: 29261409

[77] Asano N . Unveiling the anticancer effect of traditional Chinese herbal medicine. World J Gastroenterol. (2024) 30:3625–7. doi: 10.3748/wjg.v30.i30.3625, PMID: 39193575 PMC11346149

[78] Pera A Campos C López N Hassouneh F Alonso C Tarazona R . Immunosenescence: Implications for response to infection and vaccination in older people. Maturitas. (2015) 82:50–5. doi: 10.1016/j.maturitas.2015.05.004, PMID: 26044074

[79] Meng X Gao Y Yang L Jing H Teng F Huang Z . Immune microenvironment differences between squamous and non-squamous non-small-cell lung cancer and their influence on the prognosis. Clin Lung Cancer. (2019) 20:48–58. doi: 10.1016/j.cllc.2018.09.012, PMID: 30341017

[80] Chen F Li J Wang H Ba Q . Anti-tumor effects of chinese medicine compounds by regulating immune cells in microenvironment. Front Oncol. (2021) 11:746917. doi: 10.3389/fonc.2021.746917, PMID: 34722304 PMC8551633

[81] Fu K Wang C Ma C Zhou H Li Y . The potential application of chinese medicine in liver diseases: A new opportunity. Front Pharmacol. (2021) 12:771459. doi: 10.3389/fphar.2021.771459, PMID: 34803712 PMC8600187

[82] Wu L Yang FR Xing ML Lu SF Chen HL Yang QW . Multi-material basis and multi-mechanisms of the Dahuang Zhechong pill for regulating Treg/Th1 balance in hepatocellular carcinoma. Phytomedicine. (2022) 100:154055. doi: 10.1016/j.phymed.2022.154055, PMID: 35344716

[83] Wang W Zhen S Ping Y Wang L Zhang Y . Metabolomic biomarkers in liquid biopsy: accurate cancer diagnosis and prognosis monitoring. Front Oncol. (2024) 14:1331215. doi: 10.3389/fonc.2024.1331215, PMID: 38384814 PMC10879439

[84] Biselli M Conti F Gramenzi A Frigerio M Cucchetti A Fatti G . A new approach to the use of α-fetoprotein as surveillance test for hepatocellular carcinoma in patients with cirrhosis. Br J Cancer. (2015) 112:69–76. doi: 10.1038/bjc.2014.536, PMID: 25314061 PMC4453600

[85] Choi J Kim GA Han S Lee W Chun S Lim YS . Longitudinal assessment of three serum biomarkers to detect very early-stage hepatocellular carcinoma. Hepatology. (2019) 69:1983–94. doi: 10.1002/hep.30233, PMID: 30153338

[86] Wang Z Qin H Liu S Sheng J Zhang X . Precision diagnosis of hepatocellular carcinoma. Chin Med J (Engl). (2023) 136:1155–65. doi: 10.1097/CM9.0000000000002641, PMID: 36939276 PMC10278703

[87] Zhou S Wang Z Li M Wu L . Elevated preoperative serum CA125 predicts larger tumor diameter in patients with hepatocellular carcinoma and low AFP levels. BioMed Res Int. (2019) 2019:6959637. doi: 10.1155/2019/6959637, PMID: 31662990 PMC6791221

[88] Ma D Wei P Liu H Hao J Chen Z Chu Y . Multi-omics-driven discovery of invasive patterns and treatment strategies in CA19–9 positive intrahepatic cholangiocarcinoma. J Transl Med. (2024) 22:1031. doi: 10.1186/s12967-024-05854-9, PMID: 39548460 PMC11568536

[89] European Association For The Study Of The Liver; European Organisation For Research And Treatment Of Cancer . EASL-EORTC clinical practice guidelines: management of hepatocellular carcinoma. J Hepatol. (2012) 56:908–43. doi: 10.1016/j.jhep.2011 22424438

[90] Yang C Lu T Liu M Yuan X Li D Zhang J . Tiliroside targets TBK1 to induce ferroptosis and sensitize hepatocellular carcinoma to sorafenib. Phytomedicine. (2023) 111:154668. doi: 10.1016/j.phymed.2023.154668, PMID: 36657316

[91] Wu J Tang G Cheng CS Yeerken R Chan YT Fu Z . Traditional Chinese medicine for the treatment of cancers of hepatobiliary system: from clinical evidence to drug discovery. Mol Cancer. (2024) 23:218. doi: 10.1186/s12943-024-02136-2, PMID: 39354529 PMC11443773

[92] Oura K Morishita A Takuma K Nakahara M Tadokoro T Fujita K . Efficacy and outcome of molecular targeted therapies in elderly patients with hepatocellular carcinoma: Relative dose intensity associated with overall survival. Cancer Med. (2023) 12:22023–37. doi: 10.1002/cam4.6783, PMID: 38062925 PMC10757153

