# Supplementary material for: The effect of Traditional Chinese Medicine on patients undergoing targeted therapy for primary liver cancer: a systematic review and meta-analysis
Source: Front Oncol. 2025 Oct 24;15:1674965. doi: 10.3389/fonc.2025.1674965 (PMC12591985; doi:10.3389/fonc.2025.1674965)

Supplementary Material

Context:

**1 Supplementary material 1:** Search Strategy.

**2 Supplementary Table1：**Characteristics of a randomized controlled trial of oral herbal medicine combined with a target-based regimen for the treatment of PLC.

**3 Supplementary Figures**

## **Supplementary Figure 1** Subgroup analysis of quality of life.

## **Supplementary Figure 2** Subgroup analysis of AFP.

## **Supplementary Figure 3** Subgroup analysis of CA125.

## **Supplementary Figure 4** Subgroup analysis of CA199.

## **Supplementary Figure 5** Subgroup analysis of CEA.

## **Supplementary Figure 6** Subgroup analysis of ALT.

## **Supplementary Figure 7** Subgroup analysis of AST.

## **Supplementary Figure 8** Subgroup analysis of ALB.

## **Supplementary Figure 9** Subgroup analysis of TBIL.

## **Supplementary Figure 10** Subgroup analysis of CD3^+^.

## **Supplementary Figure 11** Subgroup analysis of CD4^+^.

## **Supplementary Figure 12** Subgroup analysis of CD8^+^.

## **Supplementary Figure 13** Subgroup analysis of CD4^+^/CD8^+^.

## **Supplementary Figure 14** Subgroup analysis of NK.

## **Supplementary Figure 15** Subgroup analysis of weakness.

## **Supplementary Figure 16** Subgroup analysis of loss of appetite.

## **Supplementary Figure 17** Subgroup analysis of diarrhea.

## **Supplementary Figure 18** Subgroup analysis of TCM syndrome efficacy scores.

**Supplementary Figure 19** Sensitivity analysis.

# Supplementary material 1: Search Strategy.

**Supplementary Table A: Search Strategy Used in PubMed 2025/4/20**

**Items found 18**

| No. | Search items | Items found |
| --- | --- | --- |
| #1 | (("neoplasms"[MeSH Terms] OR "carcinoma"[MeSH Terms] OR "cancer*"[Title/Abstract] OR "carcin*"[Title/Abstract] OR "neoplas*"[Title/Abstract] OR "tumo*"[Title/Abstract]) AND ("Carcinoma, Hepatocellular"[MeSH Terms] OR "liver"[MeSH Terms] OR "liver"[Title/Abstract])) | 357905 |
| #2 | ("complementary therapies"[MeSH Terms] OR "drugs, Chinese herbal"[MeSH Terms] OR "herbal medicine"[MeSH Terms] OR "medicine, traditional"[MeSH Terms] OR "medicine, east asian traditional"[MeSH Terms] OR "plant extracts"[MeSH Terms] OR "plants, medicinal"[MeSH Terms] OR "phytotherapy"[MeSH Terms] OR "alternative medicine"[Title/Abstract] OR "complementary therap*"[Title/Abstract] OR "Chinese herba*"[Title/Abstract] OR "Chinese medicine"[Title/Abstract] OR "herb*"[Title/Abstract] OR "herbalism"[Title/Abstract] OR "herbal medicine"[Title/Abstract] OR "herbal drugs"[Title/Abstract] OR "plant extract*"[Title/Abstract] OR "medicinal plant*"[Title/Abstract] OR "phytotherapy*"[Title/Abstract] OR "phytopharmaceutic*"[Title/Abstract] OR "traditional medicine"[Title/Abstract] OR "oriental medicine"[Title/Abstract] OR "zhong yi xue"[Title/Abstract]) | 616548 |
| #3 | (("randomized controlled trial"[Publication Type] OR "controlled clinical trial"[Publication Type] OR "randomized"[Title/Abstract] OR "placebo"[Title/Abstract] OR "drug therapy"[MeSH Terms] OR "randomly"[Title/Abstract] OR "trial"[Title/Abstract] OR "groups"[Title/Abstract]) NOT ("animals"[MeSH Terms] NOT "humans"[MeSH Terms])) | 4425221 |
| #4 | (("Sorafenib"[MeSH Terms] OR Donafenib[Title/Abstract] OR "lenvatinib"[Title/Abstract] OR "apatinib"[Title/Abstract] OR "regorafenib"[Title/Abstract] OR "target therapy"[Title/Abstract]) OR (Bevacizumab[Title/Abstract])) OR (Ramucirumab[Title/Abstract])) OR (Erlotinib[Title/Abstract])) OR (Cometriq[Title/Abstract])) | 34125 |
| #5 | #1 and #2 and #3 and #4 | 18 |

**Supplementary Table B. Search Strategy Used in EMBASE 2025/4/20**

**Items found 112**

| No. | Search items | Items found |
| --- | --- | --- |
| #13 | #6 AND #6 AND #9 AND #12 | 112 |
| #12 | #10 OR #11 | 3056723 |
| #11 | 'controlled clinical trial':ab,ti OR 'randomized':ab,ti OR 'placebo':ab,ti OR 'randomly':ab,ti OR 'trial':ab,ti OR 'randomization':ab,ti OR 'random':ab,ti OR 'randomize':ab,ti | 2812038 |
| #10 | 'randomized controlled trial'/exp OR 'randomized controlled trial' | 1190462 |
| #9 | #7 OR #8 | 223113 |
| #8 | 'targeted therapies':ab,ti OR 'targeted therapy':ab,ti OR 'molecular targeted therapies':ab,ti OR 'targeted molecular therapie':ab,ti OR 'sorafenib':ab,ti OR 'lenvatinib':ab,ti OR 'apatinib':ab,ti OR 'regorafenib':ab,ti OR 'erlotinib':ab,ti OR 'donafenib':ab,ti OR 'cometriq':ab,ti OR 'ramucirumab':ab,ti OR 'bevacizumab':ab,ti | 193764 |
| #7 | 'molecularly targeted therapy'/exp OR 'molecularly targeted therapy' | 66591 |
| #6 | #4 OR #5 | 355580 |
| #5 | 'zhong yi xue':ab,ti OR 'medicine, chinese traditional':ab,ti OR 'traditional chinese medicine':ab,ti OR 'complementary therapies':ab,ti OR 'herbal medicine':ab,ti OR 'alternative medicine':ab,ti OR 'herbal drugs':ab,ti OR 'phytotherapy':ab,ti OR 'phytopharmaceutic':ab,ti OR 'oriental medicine':ab,ti OR 'medicinal plant':ab,ti | 116810 |
| #4 | 'chinese medicine'/exp OR 'chinese medicine' | 298603 |
| #3 | #1 OR #2 | 418113 |
| #2 | 'liver neoplasms':ab,ti OR 'hepatic neoplasms':ab,ti OR 'liver neoplasm':ab,ti OR 'liver cancer':ab,ti OR 'hepatocellular cancer':ab,ti OR 'hepatic cancer':ab,ti | 52704 |
| #1 | 'liver tumor'/exp OR 'liver tumor' | 409435 |

**Supplementary Table C. Search Strategy Used in** **Cochrane 2025/4/20**

**Items found 25**

| No. | Search items | Items found |
| --- | --- | --- |
| #1 | MeSH descriptor: [Liver Neoplasms] explode all trees | 4547 |
| #2 | (Hepatic Neoplasms):ti,ab,kw or (Hepatic Neoplasm):ti,ab,kw or (Liver Neoplasm):ti,ab,kw or (Liver Cancer):ti,ab,kw or (Liver Cancers):ti,ab,kw or (Hepatocellular Cancer):ti,ab,kw or (Hepatocellular Cancers):ti,ab,kw or (Hepatic Cancer):ti,ab,kw or (Hepatic Cancers):ti,ab,kw or (Liver tumor):ti,ab,kw | 20212 |
| #3 | #1 or #2 | 21265 |
| #4 | MeSH descriptor: [Medicine, Chinese Traditional] explode all trees | 1798 |
| #5 | (Zhong Yi Xue):ti,ab,kw or (Chinese Traditional Medicine):ti,ab,kw or (Traditional Chinese Medicine):ti,ab,kw or (complementary therapies):ti,ab,kw or (herbal medicine):ti,ab,kw or (phytotherapy):ti,ab,kw or (alternative medicine):ti,ab,kw or (herbal drugs):ti,ab,kw or (medicinal plant):ti,ab,kw or (phytopharmaceutic):ti,ab,kw or (oriental medicine):ti,ab,kw | 28229 |
| #6 | #4 or #5 | 28430 |
| #7 | MeSH descriptor: [Molecular Targeted Therapy] explode all trees | 333 |
| #8 | (Targeted Therapy):ti,ab,kw or (targeted therapies):ti,ab,kw or (Molecular Targeted Therapies):ti,ab,kw or (Sorafenib):ti,ab,kw or (lenvatinib):ti,ab,kw or (apatinib):ti,ab,kw or (regorafenib):ti,ab,kw or (erlotinib):ti,ab,kw or (Donafenib):ti,ab,kw or (Cometriq):ti,ab,kw or (Ramucirumab):ti,ab,kw or (Bevacizumab):ti,ab,kw | 30184 |
| #9 | #7 or #8 | 30184 |
| #10 | MeSH descriptor: [Randomized Controlled Trial] explode all trees | 34 |
| #11 | (random):ti,ab,kw or (randomly):ti,ab,kw or (randomized):ti,ab,kw or (randomization):ti,ab,kw or (placebo):ti,ab,kw or (randomize):ti,ab,kw | 1480079 |
| #12 | #10 or #11 | 1480079 |
| #13 | #3 and #6 and #9 and #12 | 25 |

**Supplementary Table D. Search Strategy Used in Web of science 2025/4/20**

**Items found 2**

| No. | Search items | Items found |
| --- | --- | --- |
| #1 | (TS=("Liver Neoplasms")) OR TS=("Hepatic Neoplasms")) OR TS=("Hepatic Neoplasm")) OR TS=("Liver Neoplasm")) OR TS=("Liver Cancer")) OR TS=("Hepatocellular Cancer")) OR TS=("Hepatocellular Cancers")) OR TS=("Liver tumor")) OR TS=("Hepatic Cancer") | 51200 |
| #2 | (TS=("Medicine, Chinese Traditional")) OR TS=("Zhong Yi Xue")) OR TS=("Chinese Traditional Medicine")) OR TS=("Traditional Chinese Medicine")) OR TS=("complementary therapies")) OR TS=("herbal medicine")) OR TS=("phytotherapy")) OR TS=("alternative medicine")) OR TS=("herbal drugs")) OR TS=("medicinal plant")) OR TS=("phytopharmaceutic")) OR TS=("oriental medicine") | 112465 |
| #3 | (TS=("Molecular Targeted Therapy")) OR TS=("Targeted Therapy")) OR TS=("targeted therapies")) OR TS=("Sorafenib")) OR TS=("lenvatinib")) OR TS=("apatinib")) OR TS=("regorafenib")) OR TS=("erlotinib")) OR TS=("Donafenib")) OR TS=("Cometriq")) OR TS=("Ramuciruma")) OR TS=("Bevacizumab") | 162427 |
| #4 | (TS=("randomized controlled trial")) OR TS=("random")) OR TS=("randomly")) OR TS=("randomized")) OR TS=("randomization")) OR TS=("placebo")) OR TS=("randomize") | 2392979 |
| #5 | #1 AND #2 AND #3 AND #4 | 2 |

**Supplementary E: Search Strategy Used in CNKI 2025/4/20**

**Items found 69**

（主题：肝肿瘤）OR（主题：肝部肿瘤）OR（主题：肝细胞癌症）OR（主题：肝细胞癌）OR（主题：肝癌）OR（主题：肝恶性肿瘤）OR（主题：原发性肝细胞癌）OR（主题：肝脏恶性肿瘤）OR（篇关摘：肝脏肿瘤(精确)）AND（主题：中草药）OR（主题：中医）OR（主题：中医药）OR（主题：中药材）OR（主题：传统医学）OR（主题：补充替代医学）OR（主题：汤药）OR（主题：方）OR（主题：煎剂）OR（主题：散）AND（主题：分子靶向治疗）OR（主题：多纳非尼）OR（主题：仑伐替尼）OR（主题：阿帕替尼）OR（主题：索拉非尼）OR（主题：安罗替尼）OR（主题：瑞戈非尼）OR（主题：卡博替尼）OR（主题：雷莫西尤单抗）OR（主题：贝伐珠单抗）AND（主题：随机对照试验）OR（主题：随机）OR（主题：RCT）OR（主题：临床观察）OR（主题：疗效）OR（主题：临床研究）OR（主题：临床效果）OR（主题：评价）OR（主题：应用）OR（主题：体会）

**Supplementary F: Search Strategy Used in WangFang 2025/4/20**

**Items found 2965**

主题:(肝肿瘤 or 肝脏肿瘤 or 肝细胞癌 or 肝癌 or 肝脏肿瘤 or 肝恶性肿瘤 or 原发性肝细胞癌 or 肝脏恶性肿瘤) and 主题:(中草药 or 中药 or 中医 or 中医药 or 中药材 or 植物药 or 传统医学 or 汤药 or 方 or 丸 or 法 or 散 or 煎剂) and 主题:(分子靶向治疗 or 仑伐替尼 or 阿帕替尼 or 索拉非尼 or 安罗替尼 or 瑞戈非尼 or 多纳非尼 or 卡博替尼 or 雷莫西尤单抗 or 贝伐珠单抗) and 主题:(随机对照试验 or 随机 or RCT or 临床观察 or 临床效果 or 临床研究 or 疗效 or 评价 or 应用 or 体会)

**Supplementary G: Search Strategy Used in CBM 2025/4/20**

**Items found 35**

("随机对照试验"[不加权:扩展]) AND (("仑伐替尼"[常用字段:智能] OR "阿帕替尼"[常用字段:智能] OR "索拉非尼"[常用字段:智能] OR "贝伐珠单抗"[常用字段:智能] OR "靶向治疗"[常用字段:智能] OR "安罗替尼"[常用字段:智能] OR "多纳非尼"[常用字段:智能] OR "卡博替尼"[常用字段:智能] OR "雷莫西尤单抗"[常用字段:智能]) OR ("分子靶向治疗"[不加权:扩展])) AND (("中医药"[常用字段:智能] OR "中药"[常用字段:智能] OR "中医"[常用字段:智能] OR "中药材"[常用字段:智能] OR "植物药"[常用字段:智能] OR "传统医学"[常用字段:智能] OR "汤药"[常用字段:智能] OR "方"[常用字段:智能] OR "散"[常用字段:智能]) OR ("中草药"[不加权:扩展])) AND (("肝部肿瘤"[常用字段:智能] OR "肝脏肿瘤"[常用字段:智能] OR "肝细胞癌症"[常用字段:智能] OR "肝细胞癌"[常用字段:智能] OR "肝癌"[常用字段:智能] OR "肝脏肿瘤"[常用字段:智能] OR "肝恶性肿瘤"[常用字段:智能] OR "原发性肝细胞癌"[常用字段:智能] OR "肝脏恶性肿瘤"[常用字段:智能]) OR ("肝肿瘤"[不加权:扩展]))

# Supplementary Table1：Characteristics of a randomized controlled trial of oral herbal medicine combined with a target-based regimen for the treatment of PLC.

| **Ref.** | **Design** | **Sample size T/C;** | **Sex**  **(Male and Female)** | | **Combination therapy group intervention** | **Control group intervention** | **Outcome measures** |
| --- | --- | --- | --- | --- | --- | --- | --- |
|  |  | **Age T/C** | **T** | **C** |  |  |  |
| Zou ZC  *et al*., 2023 | RCT | 30/30;  56.72±5.64/  56.71±5.76 | 22/8 | 23/7 | Yin-Yang Gongji Wan  po | Lenvatinib+Sindilizumab ：  Lenvatinib po qd; Body mass <60 kg, 8 mg/d; body mass ≥60 kg, 12 mg/d.  Sindilizumab, 200 mg，ivgtt; 3 weeks/cycle. | O6,8,9 |
| Ren J  *et al*.,2024 | RCT | 40/40;  57.16±10.23/  56.82±9.39 | 28/12 | 26/14 | Yiqi Jiedu Formula 200ml po bid; Lasts 8 weeks. | Apatinib 750mg po qd，for 8 weeks. | O1,7,8 |
| Wu WY  *et al*., 2023 | RCT | 43/43;  64.46±3.52/  63.57±3.43 | 22/21 | 23/20 | Yiqi Huayu Jiedu Decoction 100ml po bid; 3 weeks/cycl，for 2 cycles. | Sorafenib+Tegafur:Sorafenib 0.4g po bid; Tegafur 40-60 mg po bid；2 weeks of treatment, 1 week off，3 weeks/cycle，for 2 cycles. | O1,8 |
| Kong KK  *et al*.,2020 | RCT | 25/25;  62.12±3.72/  61.48±5.45 | 17/8 | 18/7 | Yiqi Huayu Jiedu Decoction 100ml po bid; for 6 weeks. | Sorafenib+Tegafur: Sorafenib 400mg po bid; Tegafur 40-60 mg po bid；2 weeks of treatment, 1 week off，3 weeks/cycle，for 2 cycles. | O1,3 |
| Zhang Z  *et al*.,2019 | RCT | 28/28;  55.93±7.03/  56.68±6.56 | 20/8 | 18/10 | Yiqi Huayu Jiedu Formula po bid. | Sorafenib 400mg po bid. | O2,3,8 |
| Zhao D  *et al*., 2018 | RCT | 34/30;  55.36/55.13 | 25/9 | 20/10 | Yiqi Yanggan Formula 200ml po bid. | Sorafenib 400mg po bid. | O1,2,8 |
| Li YF  *et al*., 2024 | RCT | 32/30;  54.75±8.03/  56.30±8.93 | 22/10 | 19/11 | Yiguanjian Plus 150ml po bid; 30days/cycle, for 2 cycles. | Lenvatinib po qd; Body mass <60 kg, 8 mg/d; body mass ≥60 kg, 12 mg/d. 30days/cycle, for 2 cycles. | O1,2,4,5,8,9 |
| Cao SM  *et al*.,2022 | RCT | 28/27;  58.46±12.20/58.93±12.53 | 20/8 | 17/10 | Yangzheng Xiaoji Capsule 4 grains，po tid. 21days/cycles;for 4 cycles. | Apatinib +Karelizumab: Apatinib 250mg po qd; Karelizumab200mg，ivgtt, 3 weeks/session. | O1,2,6,8,9 |
| Yao SS *et al*.,2023 | RCT | 50/50;  54.74±7.15/  54.82±7.26 | 30/20 | 28/22 | Xuan Yu Hua Du Decoction 200ml po bid; 21days/cycles. for 4 cycles. | Sorafenib 400mg po bid. 21days/cycles. for 4 cycles. | O1,4,5,7,  8,9 |
| Ding W  *et al*., 2024 | RCT | 32/32;  58.66±11.24/60.56±9.47 | 23/9 | 21/11 | Xiaoyao San Plus 150ml po bid. | Lenvatinib po qd; Body mass <60 kg, 8 mg/d; body mass ≥60 kg, 12 mg/d. 28days/cycle, for 3 cycles. | O1,2,4,5,8,9 |
| Gai JZ  *et al*., 2024 | RCT | 30/30;  61.77±9.30/  63.70±9.57 | 18/12 | 16/14 | Shugan Jianpi Huazhuo Formula 150ml po bid. | Sorafenib 0.4g po bid; 4weeks/cycles; for 2 cycles. | O1,2,4,5,7,8,9 |
| Duan KN  *et al*., 2018 | RCT | 23/22;  65.66±2.12/  65.70±2.23 | 17/6 | 15/7 | Discriminative Chinese Medicine Soup  po 1 dose daily. | Sorafenib 400mg po bid; for 2 months. | O1,2 |
| Wan XY  *et al*., 2022 | RCT | 31/31;  58.26±9.23/  57.93±9.18 | 22/9 | 23/8 | Jiawei Xiaoyao San 100ml po bid. | Sorafenib 400mg po bid; for 3 weeks. | O1,8 |
| Huang SY  *et al*.,2023 | RCT | 24/24;  58.17 ± 7.18/  58.12 ± 7.25 | 15/9 | 17/7 | Siteng Fang + Yinchenhao Decoction Modification po bid; for 30 days. | Lenvatinib po qd; Body mass <60 kg, 8 mg/d; body mass ≥60 kg, 12 mg/d. | O1,8,9 |
| Huang PP  *et al*., 2024 | RCT | 30/30;  65.9±8.8/  66.6±12.4 | 25/5 | 25/5 | Sini Tang Granules 1pack po tid; 3weeks/cycle; for 4 cycles. | Lenvatinib+Sindilizumab:  Lenvatinib po qd; Body mass <60 kg, 8 mg/d; body mass ≥60 kg, 12 mg/d.  Sindilizumab, 200 mg，ivgtt; 3 weeks/cycle. | O2,4,6,8,9 |
| Zhou K  *et al*., 2024 | RCT | 31/30;  63.79±3.40/  63.82±3.38 | 18/13 | 18/12 | Shugan Jianpi Jiedu Formula 150ml po tid. | Tislelizumab+Bevacizumab;Tislelizumab 200mg ivgtt Bevacizumab 15mg/kg, 21d/session,for 3 months. | O1,2,4,5,6,8,9 |
| Yang JL  *et al*., 2021 | RCT | 35/35;  51.29±2.37/  51.16±2.45 | 24/11 | 23/12 | Si Jun Zi Tang po bid, for 2 months. | Regorafenib:80-120mg po qd, 3 weeks of treatment, 1 week off, 4 weeks/cycle,for 2 cycles. | O1,6,8 |
| Mei MR  *et al*., 2024 | RCT | 30/30;  50.16±7.62/  49.90±8.99 | 21/9 | 22/8 | Rougan Sanjie Wan 10g po tid. | Lenvatinib po qd; Body mass <60 kg, 8 mg/d; body mass ≥60 kg, 12 mg/d. for 12 weeks. | O1,4,5,7,8,9 |
| Liu SM  *et al*., 2024 | RCT | 55/55;  73.51±4.96/  73.87±4.98 | 39/16 | 40/15 | Qinggan Xiaozheng Formula Modification 150 ml，po bid; 3 weeks/cycles; for 4 cycles. | Lenvatinib+Camrelizuma: Lenvatinib po qd; Body mass <60 kg, 8 mg/d; body mass ≥60 kg, 12 mg/d; Camrelizumab 200mg ivgtt; 3 weeks/cycles; for 4 cycles. | O1,4,6,8 |
| Zhu WL  *et al*., 2022 | RCT | 35/35;  49.94±11.35/51.60±11.07 | 33/2 | 28/7 | Qi'e Baogan Formula 150ml po bid 1month/cycle. | Lenvatinib po qd; Body mass <60 kg, 8 mg/d; body mass ≥60 kg, 12 mg/d. | O4,5,8,9 |
| Li SD  *et al*.,2023 | RCT | 32/33;  60.56±8.79/  61.61±11.53 | 22/10 | 28/5 | Poyu Jiedu Formula po bid. | Lenvatinib po qd; Body mass <60 kg, 8 mg/d; body mass ≥60 kg, 12 mg/d.for 12 weeks. | O1,2,4,5,7,8,9 |
| Wei GP  *et al*., 2025 | RCT | 46/43;  59.03 ±6.10/  57.78±5.24 | 31/14 | 27/16 | Peiyuan Guben Formula 150ml po bid; 2 weeks of treatment, 1 week off, for 6 cycles. | Anlotini 12mg po qd; 2 weeks of treatment, 1 week off, for 6 cycles. | O1,4,5,6 |
| Tu XL  *et al*., 2021 | RCT | 30/30;  53.93±5.29/  53.56±6.12 | 19/11 | 18/12 | Jianpi Yanggan Jiedu Formula 150ml po bid;14 days/cycles;for 4 cycles. | Lenvatinib po qd; Body mass <60 kg, 8 mg/d; body mass ≥60 kg, 12 mg/d.14 days/cycles;for 4 cycles. | O1,2,9 |
| Wu YW  *et al*.,2018 | RCT | 30/30;  58.04±11.41/60.8±14.11 | 27/3 | 28/2 | Jianpi Rougan Formula 125ml po bid; 28 days/cycles;At least 1 cycles. | Apatinib 250mg，po qd. 28 days/cycles;At least 1 cycles. | O1,2,4,8,9 |
| Ma YK  *et al*., 2018 | RCT | 20/20;  58.8±7.7/  59.6±9.3 | 17/3 | 15/5 | Jianpi Jiedu Formula150ml po bid. | Apatinib 250mg，po qd. 4 weeks/cycles;for 2 cycles. | O1,4,6,8 |
| Ye JH  *et al*., 2022 | RCT | 20/20;  61.50±4.32/  62.10±7.06 | 9/11 | 11/9 | Jianpi Jiedu  Formula 200ml po bid. | Lenvatinib+Camrelizuma: Lenvatinib po qd; Body mass <60 kg, 8 mg/d; body mass ≥60 kg, 12 mg/d; Camrelizumab 3mg/kg ivgtt; 14 weeks/cycles; for 2 cycles. | O1,2,4,6,9 |
| Sun Y  *et al*.,2019 | RT | 30/30;  65.69±1.38/65.53±1.25 | 17/13 | 16/14 | Jiawei Yiguanjian 120ml po bid. | Apatinib 500mg，po qd. | O1 |
| Yang CJ  *et al*.,2021 | RCT | 27/26;  56.96±9.89/  54.88±10.25 | 19/8 | 20/6 | Jiawei Xiaochaihu Tang 1cell po tid. | Sorafenib 400mg po bid; 1 month/cycle;for 2 cycles. | O1,2,4,5,8,9 |
| Li JY  *et al.*,2024 | RCT | 40/40;  57.21±4.75/57.51±4.86 | 21/19 | 19/21 | Jianpi Xingqi  Jiedu Therapy100ml po bid. | Sorafenib +radiotherapy:Sorafenib 0.4g po bid; Radiotherapy doses of 30-60 Gy, set at 2 Gy as a split dose.for 4 weeks. | O4,5,8,9 |
| Liu JP  *et al*.,2018 | RCT | 33/33;  56.6±5.5/  56.9±5.5 | 16/17 | 18/15 | Qinghuo Tongluo Formula 200ml po tid. | Sorafenib 0.4g po bid; for 3 months. | O1,2,4,5,8 |
| Jin Z  *et al*., 2022 | RCT | 29/29;  56.7±4.6/  56.4±4.3 | 18/12 | 20/10 | Huisheng Oral Liquid  10ml po tid. | Lenvatinib po qd; Body mass <60 kg, 8 mg/d; body mass ≥60 kg, 12 mg/d; for 8 weeks. | O1,4,8 |
| Han DZ  *et al*.,2022 | RCT | 57/57;  46.57±1.34/  46.60±1.41 | 37/20 | 39/18 | Huaier Granule  20g po tid. | Sorafenib 0.4g po bid; 1 month/cycles;for 2 cycles. | O1,4,8 |
| Tang YF  *et al*.,2018 | RCT | 57/56;  51.97±7.41/  52.34±7.27 | 40/17 | 41/15 | Huaier Granule  20g po tid. | Sorafenib 0.4g po bid; for 2 months. | O1,2,4,5,7,8 |
| Zhang QH *et al*.,2019 | RCT | 30/30;  49.63 ± 9.06/  52.57 ± 8.92 | 23/7 | 21/9 | Huaier Granule  20g po tid. | Sorafenib 0.4g po bid;1 month/cycles;for 2 cycles. | O1,8 |
| Ma JR  *et al*.,2022 | RCT | 54/54;  54.96±9.15/  55.31±9.20 | 30/25 | 32/23 | Huazhi Rougan  Granule  8g po tid;for 3 months. | Regorafenib:160mg po qd, 28 days /cycles;for 3 months. | O1,5,8,9 |
| Ma J  *et al*., 2024 | RCT | 33/34;  60.60±4.06/  61.62±4.08 | 23/10 | 25/9 | Fufang Shougong Powder  2.5g po bid. | Lenvatinib po qd; Body mass <60 kg, 8 mg/d; body mass ≥60 kg, 12 mg/d; 28 days/cycles;for 2 cycles. | O1,4,6,7,8,9 |
| Yu JF  *et al*.,2021 | RCT | 33/32;  47.33±12.35/48.82±12.34 | 27/6 | 27/5 | Fuling Sini  Formula po. | Sorafenib 400mg po bid;At least 4 weeks. | O1,6 |
| Chen WZ  *et al*., 2024 | RCT | 42/42;  63.52±4.36/  63.49±4.28 | 32/10 | 33/9 | Fuling Sini  Formula  200ml po bid. | Apatinib+Camrelizuma: Apatinib250-500mg po qd; Camrelizumab 200mg ivgtt; 3 weeks/cycles; for 3 months. | O1,4,6,8 |
| Liu WQ  *et al*., 2024 | RCT | 34/32;  64.97±9.74/  66.44±7.63 | 24/10 | 19/13 | Fuzheng Sanjie Formula  150ml po bid. | Apatinib250mg po qd; 3 weeks/cycles;for 2 cycles. | O1,2,4,6,8,9 |
| Liang F  *et al*., 2024 | RCT | 50/50;  56.73±10.15/56.83±10.27 | 30/20 | 29/21 | Fuzheng Anzhong Formula  100ml po bid. | Sorafenib 400mg po bid; 2 months/cycles; for 3 cycles. | O1,3,4,5,7,8,9 |
| Zhong XT  *et al*., 2023 | RCT | 50/50;  53.4±6.2/  54.7±7.8 | 28/22 | 30/20 | Fuhe Beihua  Formula  200ml po tid. | Apatinib+Camrelizuma: Apatinib 0.25g po qd; Camrelizumab 200mg ivgtt; 3 weeks/cycles. | O1,2,4,6,8 |
| Kong DC  *et al*., 2020 | RCT | 28/28;  58.13±5.79/  57.63±5.82 | 16/12 | 15/13 | Yiqi Huayu  Jiedu  Formula  200-300ml po bid. | Sorafenib 0.4g po bid. | O2,3 |
| Zhu X  *et al*., 2023 | RCT | 26/26;  60.00±9.453/61.38±9.192 | 21/5 | 22/4 | Danzhi Xiaoyao San Plus  150ml po bid. | Lenvatinib po qd; Body mass <60 kg, 8 mg/d; body mass ≥60 kg, 12 mg/d; 30 days/cycles;for 2 cycles. | O1,2,4,8,9 |
| Zhan LH  *et al*., 2022 | RCT | 30/30;  58.83±10.93/58.8±12.93 | 18/12 | 21/9 | Xiaochaihu Tang plus Danggui Shaoyao San Granules  150ml po bid. | Apatinib 0.25g po qd; 4 weeks/cycles; for 3 cycles. | O1,2,4,5,8,9 |
| Xu WC  *et al*., 2023 | RCT | 30/30;  54.6±9.18/  57.73±11.02 | 21/9 | 24/6 | Chaiping Formula 100ml po bid;4 weeks/cycles; for 3 cycles. | Lenvatinib+Camrelizuma: Lenvatinib po qd; Body mass <60 kg, 8 mg/d; body mass ≥60 kg, 12 mg/d; 4 weeks/cycles; for 3 cycles. Camrelizumab 3mg/kg ivgtt; 4 weeks/cycles; for 4 cycles. | O1,2,4,5,8,9 |
| Jiang XQ  *et al*., 2022 | RCT | 37/38;  61.21±4.28/  61.28±4.30 | 20/17 | 22/16 | Chaihu Shugan Huayu Formula  200ml po bid. | Lenvatinib po qd; Body mass <60 kg, 8 mg/d; body mass ≥60 kg, 12 mg/d; 21 days/cycles;for 3 cycles. | O1,2,4,5,9 |
| Han GM  *et al*., 2021 | RCT | 29/29;  53.36±10.25/53.73±10.41 | 10/19 | 13/16 | Chaihu Biejia  Formula  po bid. | Sorafenib 0.4g po bid; for 2 months. | O1,2,3 |
| Fang HS  *et al*., 2015 | RCT | 30/30;  59.5±11.23/57.9±10.59 | 17/13 | 14/16 | Shentao Ruangan Formula  150-200ml po. | Sorafenib 400mg po bid; 4 weels/cycles. | O1,2,3,4,5,8 |
| Wang X  *et al*., 2025 | RCT | 26/26;  66.69±7.10/  68.31±6.87 | 20/6 | 18/8 | Shenqi Xiaoji  Formula  150ml po bid; for 4 cycles. | Lenvatinib +Tislelizumab:Lenvatinib po qd; Body mass <60 kg, 8 mg/d; body mass ≥60 kg, 12 mg/d; 4  Tislelizumab  200mg ivgtt; 21weeks/cycles; for 4 cycles. | O1,2,4,8,9 |

**Abbreviations:** T, treatment group; C, control group; qd, once a day; bid, twice per day; tid, three per day; C, control group; d, day; ivgtt, injection venosa gutta; O: outcomes;O1:Clinical efficacy O2:Karnofsky Performance Status(KPS) O3:1-year survival rate O4: Tumor markers O5:Liver function O6:Immune function-related indexes O7:Inflammation-related indexes O8:Adverse drug reactions O9:TCM evidence score.

## 3 Supplementary Figures

## **Supplementary Figure 1:** Subgroup analysis of quality of life.

##
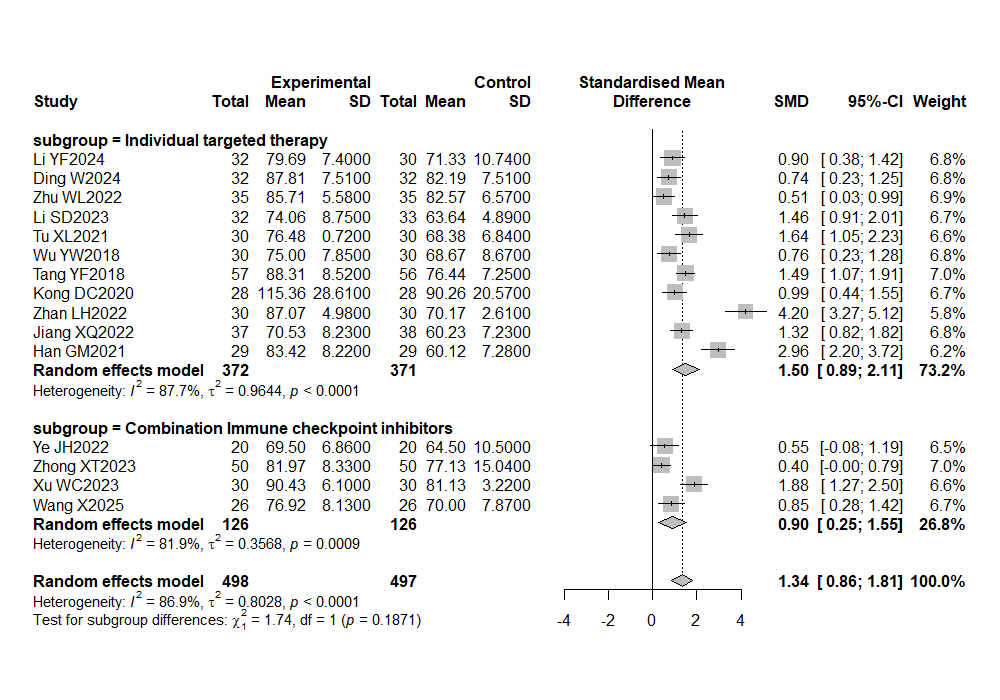


**Supplementary Figure 2:** Subgroup analysis of AFP.


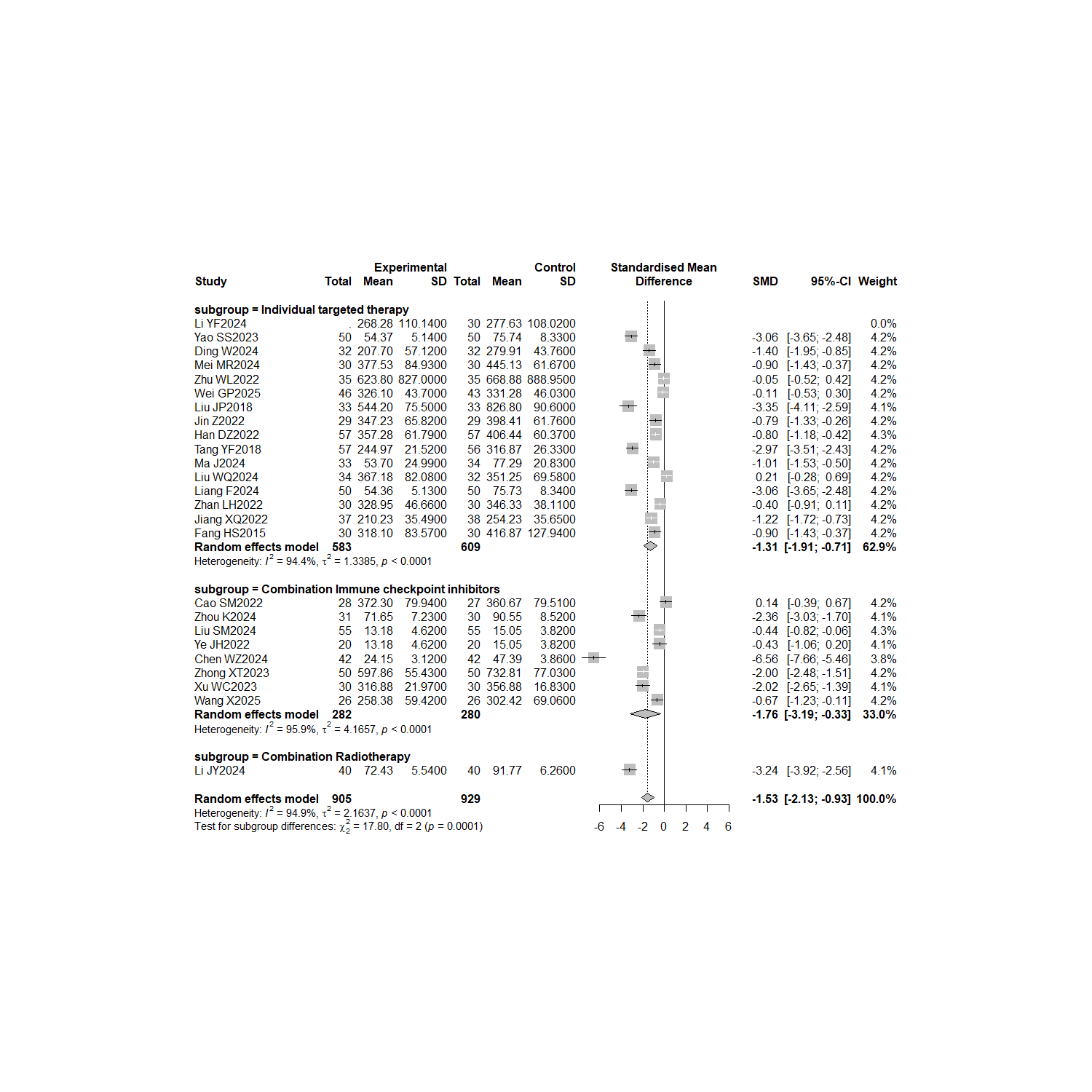


**Supplementary Figure 3:** Subgroup analysis of CA125.


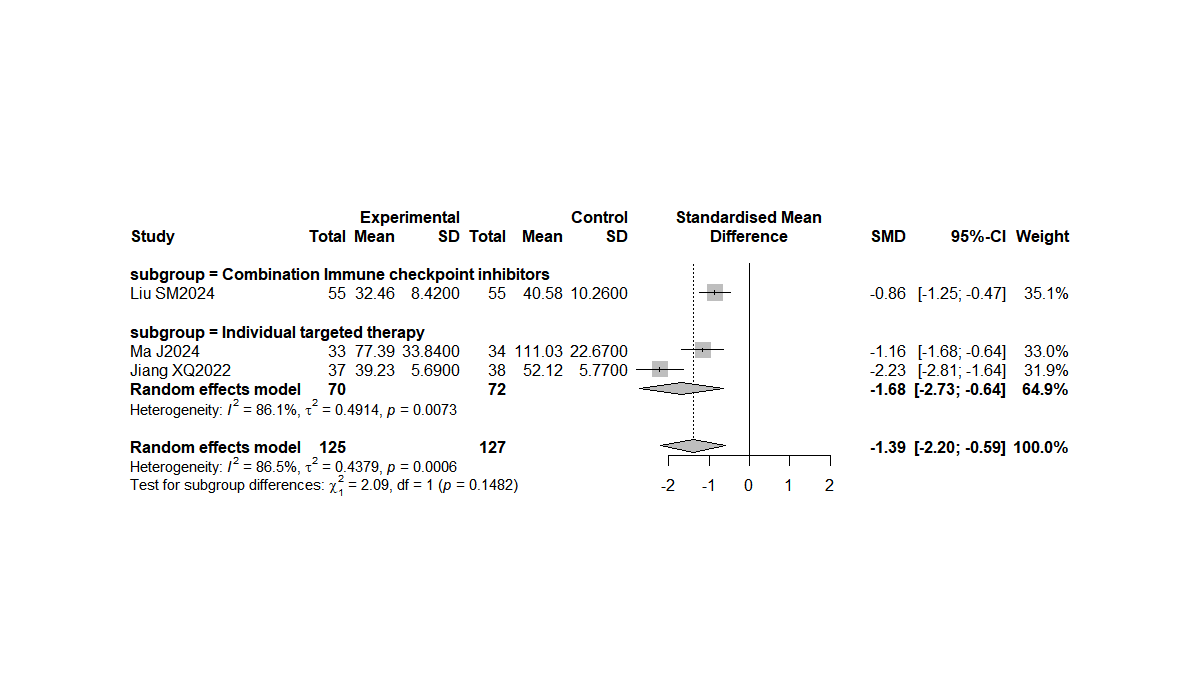


**Supplementary Figure 4:** Subgroup analysis of CA199.


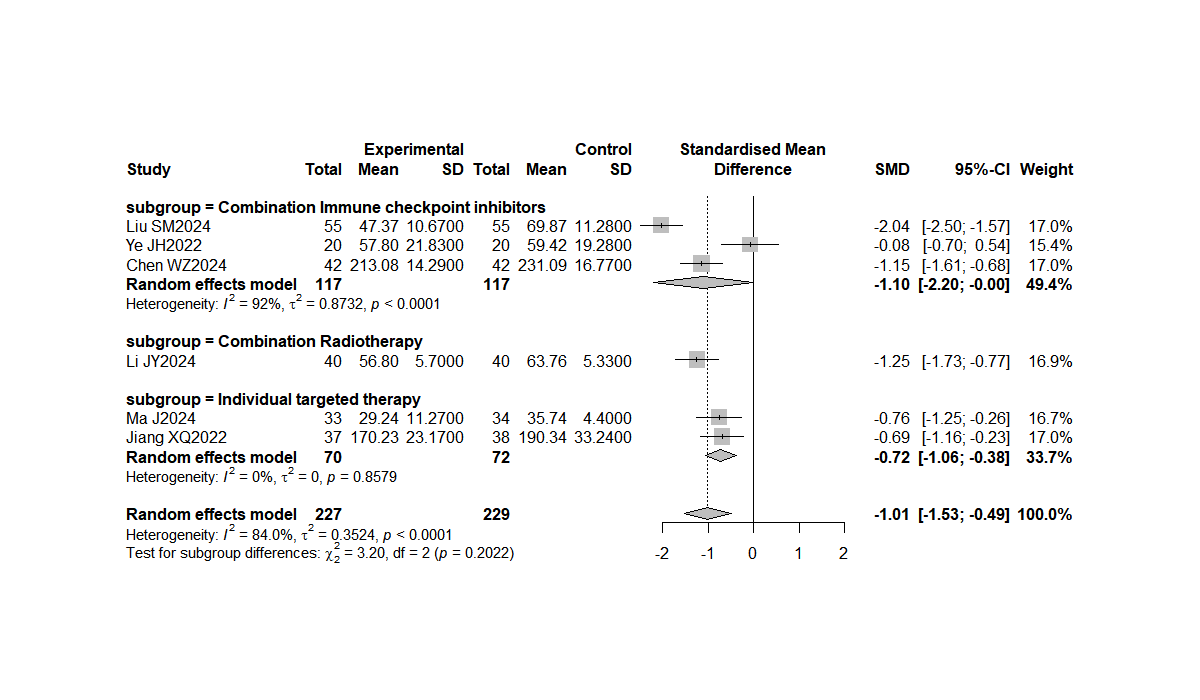


**Supplementary Figure 5:** Subgroup analysis of CEA.


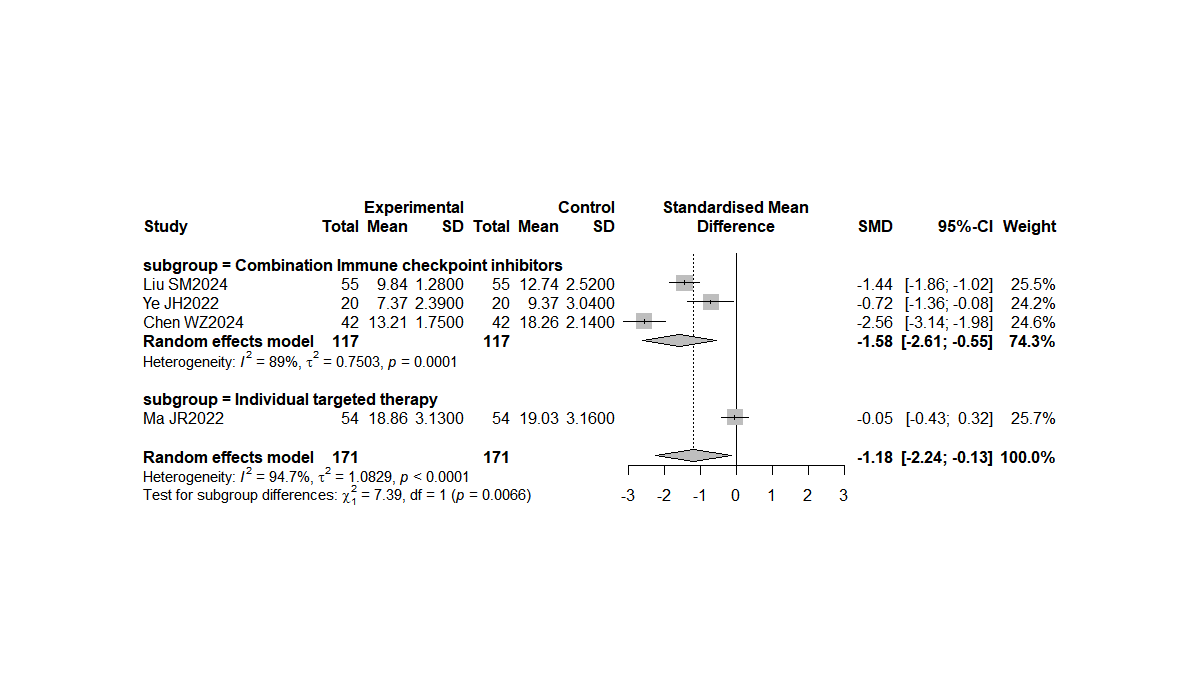


**Supplementary Figure 6:** Subgroup analysis of ALT.


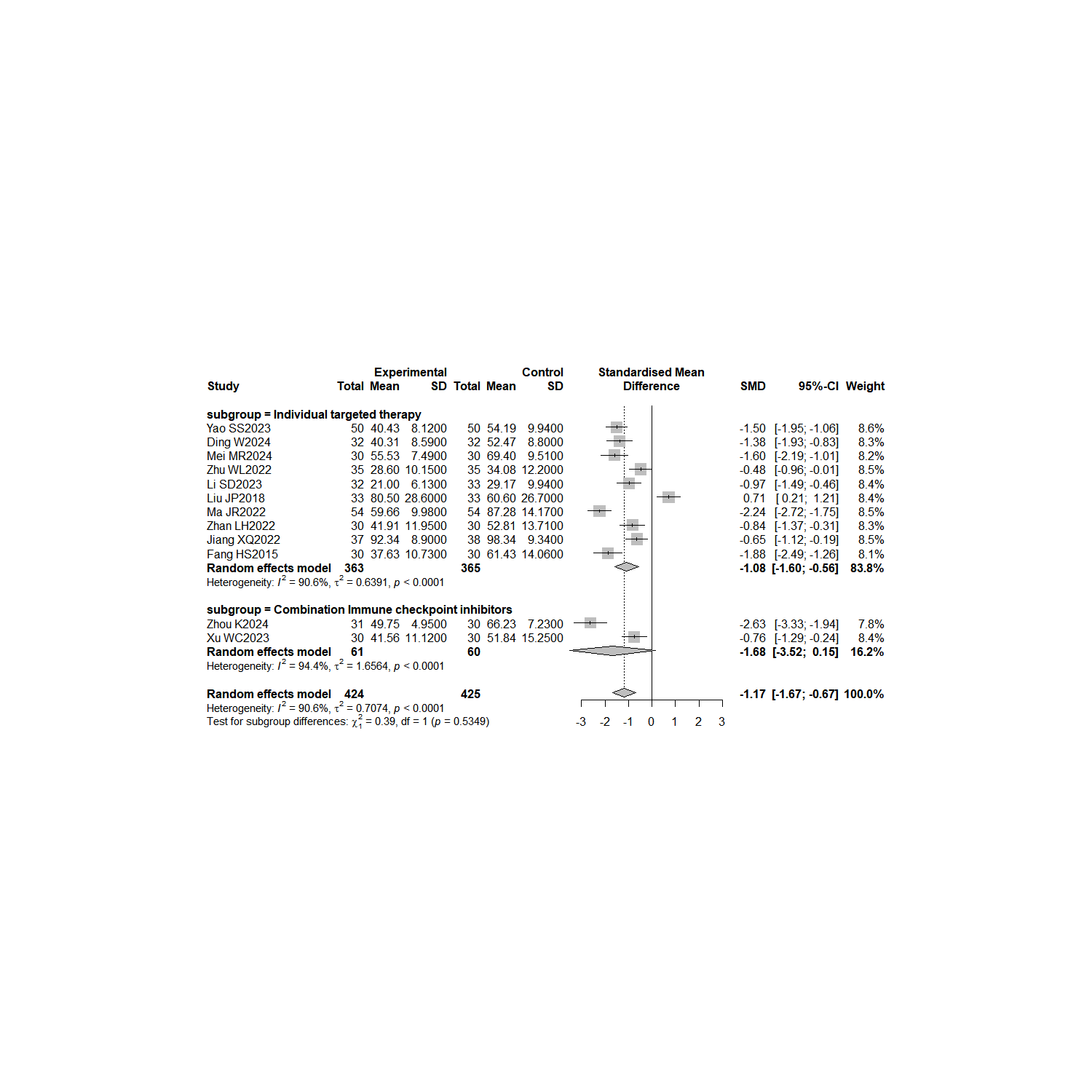


**Supplementary Figure 7:** Subgroup analysis of AST.


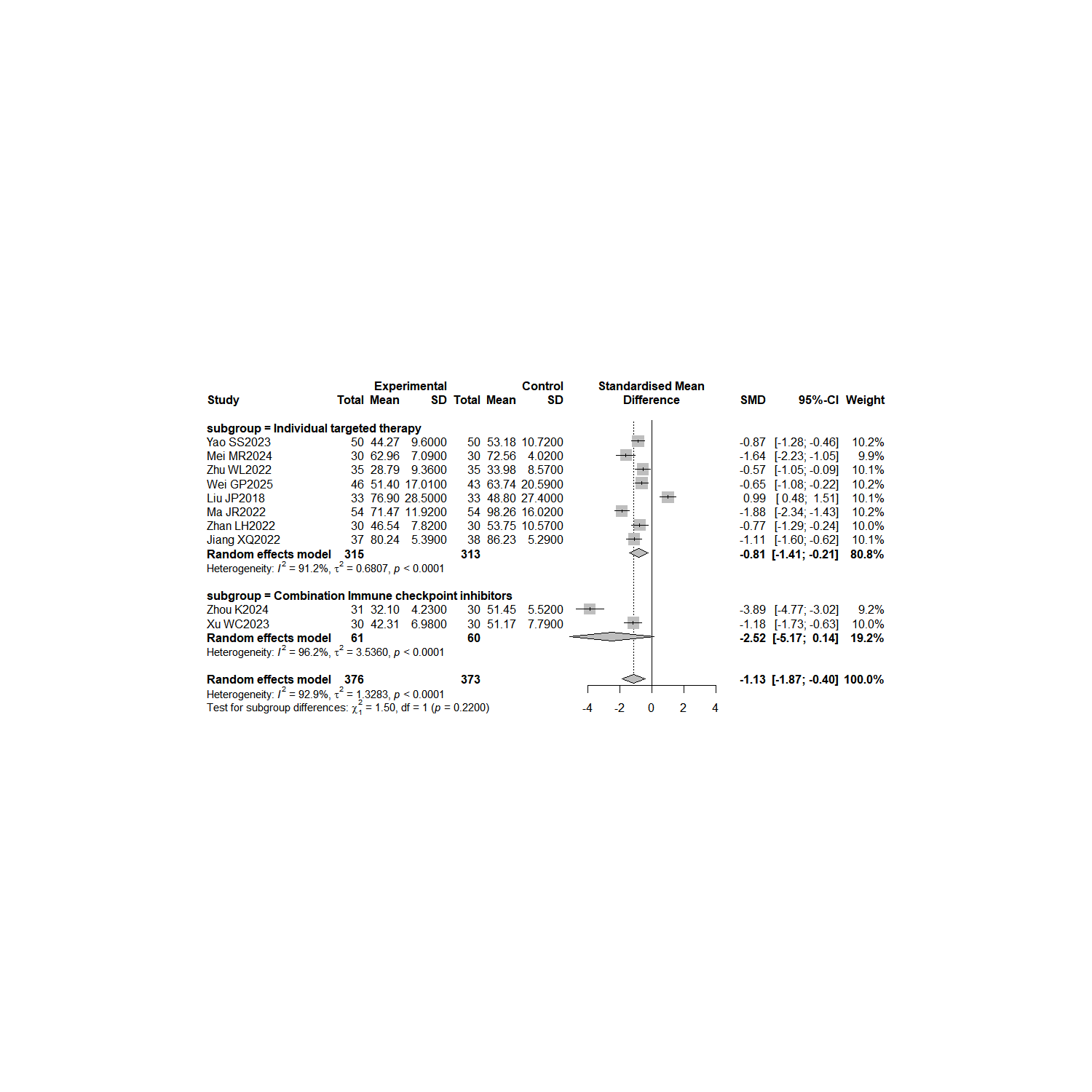


**Supplementary Figure 8:** Subgroup analysis of ALB.


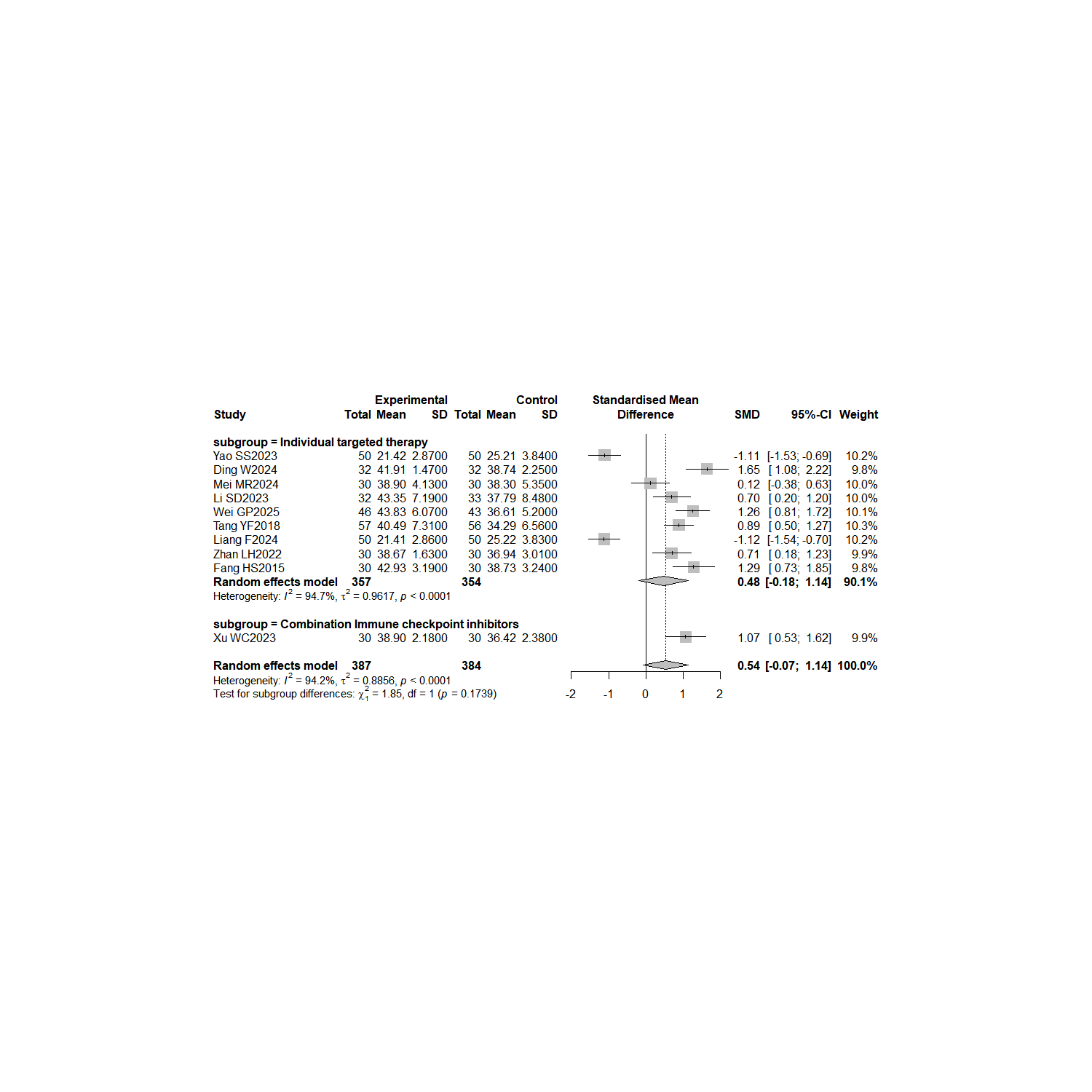


**Supplementary Figure 9:** Subgroup analysis of TBIL.


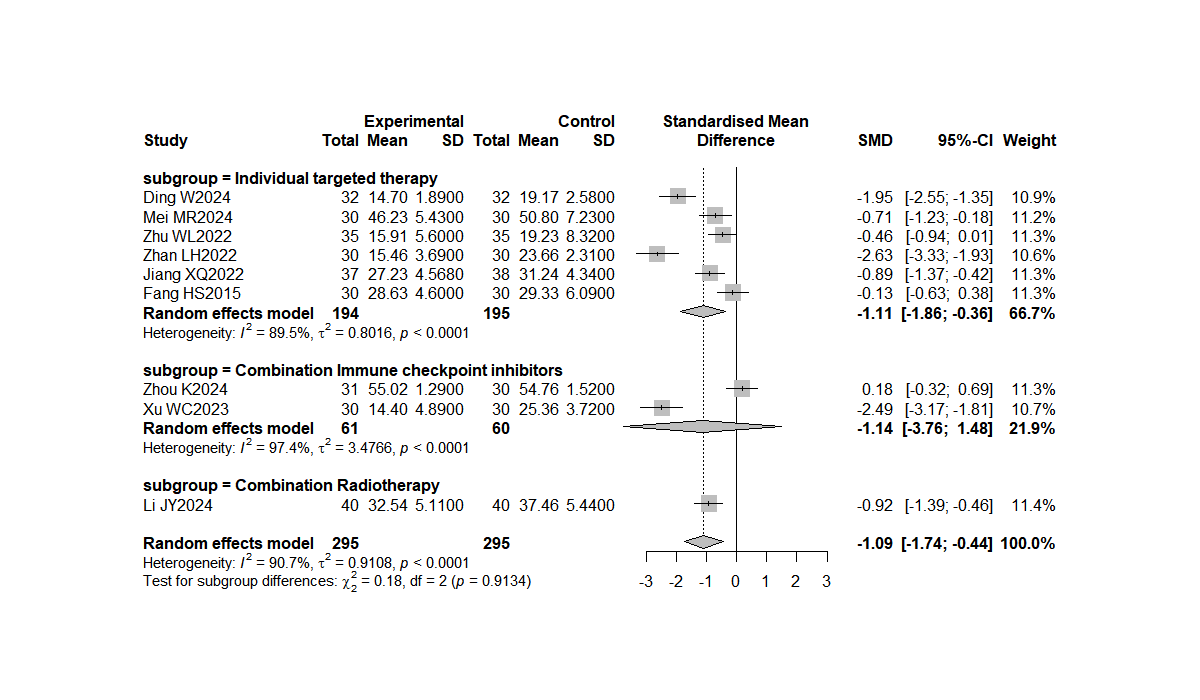


**Supplementary Figure 10:** Subgroup analysis of CD3^+^.


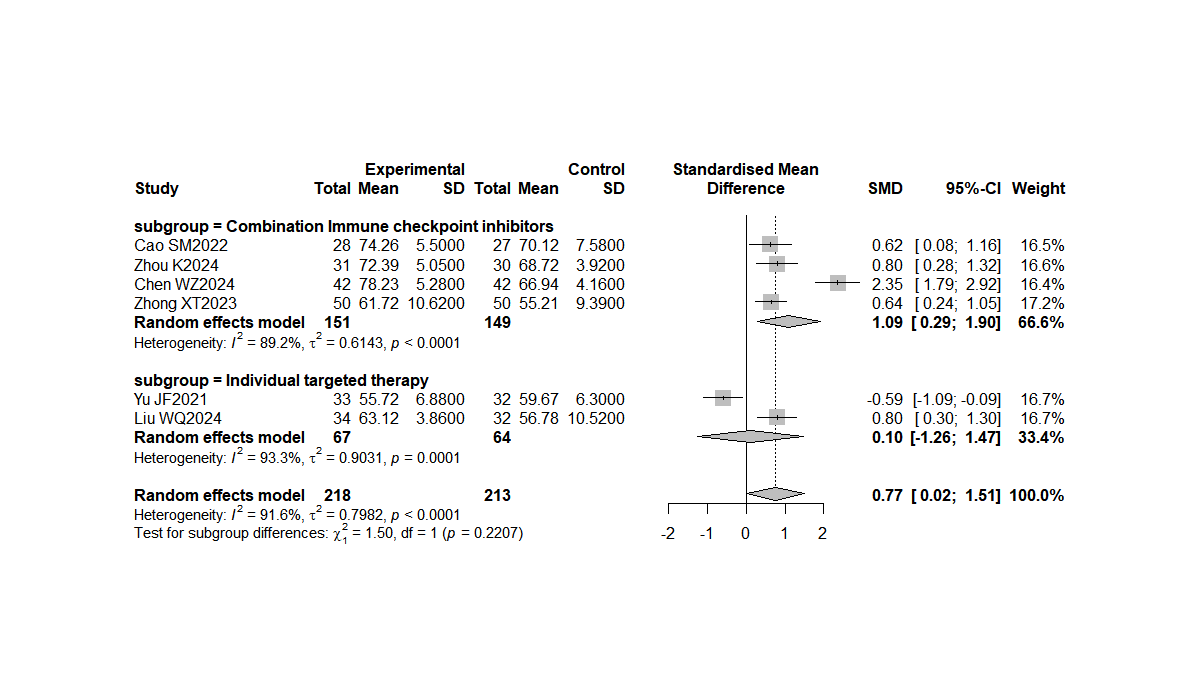


**Supplementary Figure 11:** Subgroup analysis of CD4^+^.


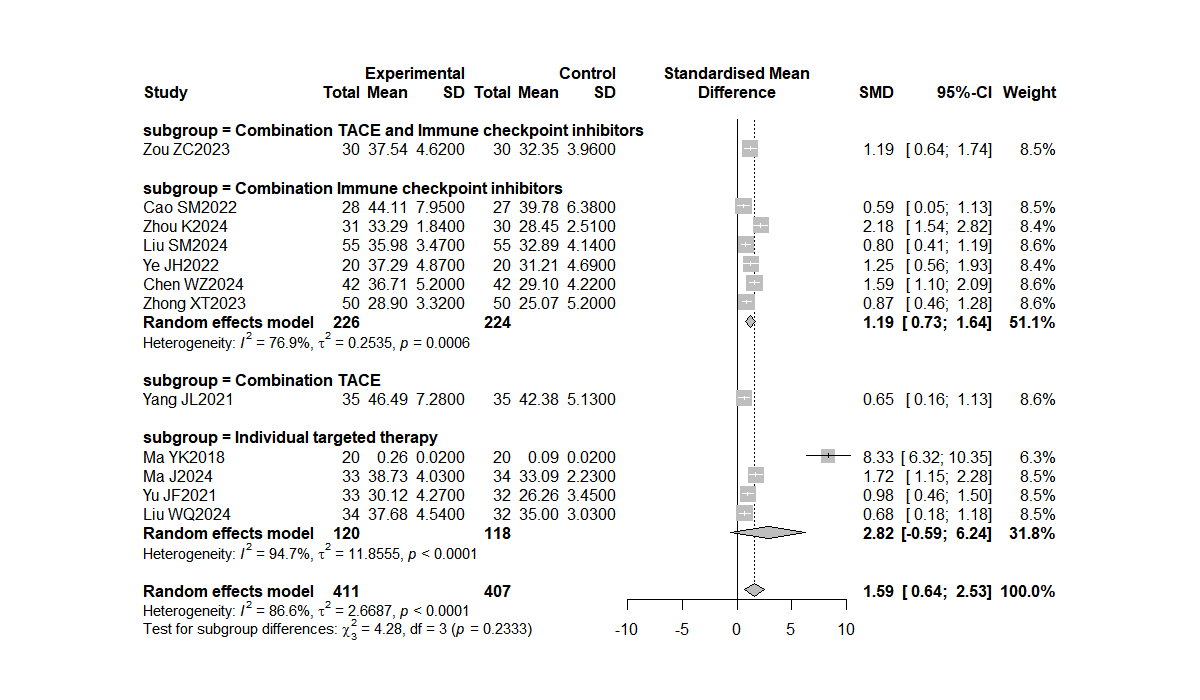


**Supplementary Figure 12:** Subgroup analysis of CD8^+^.


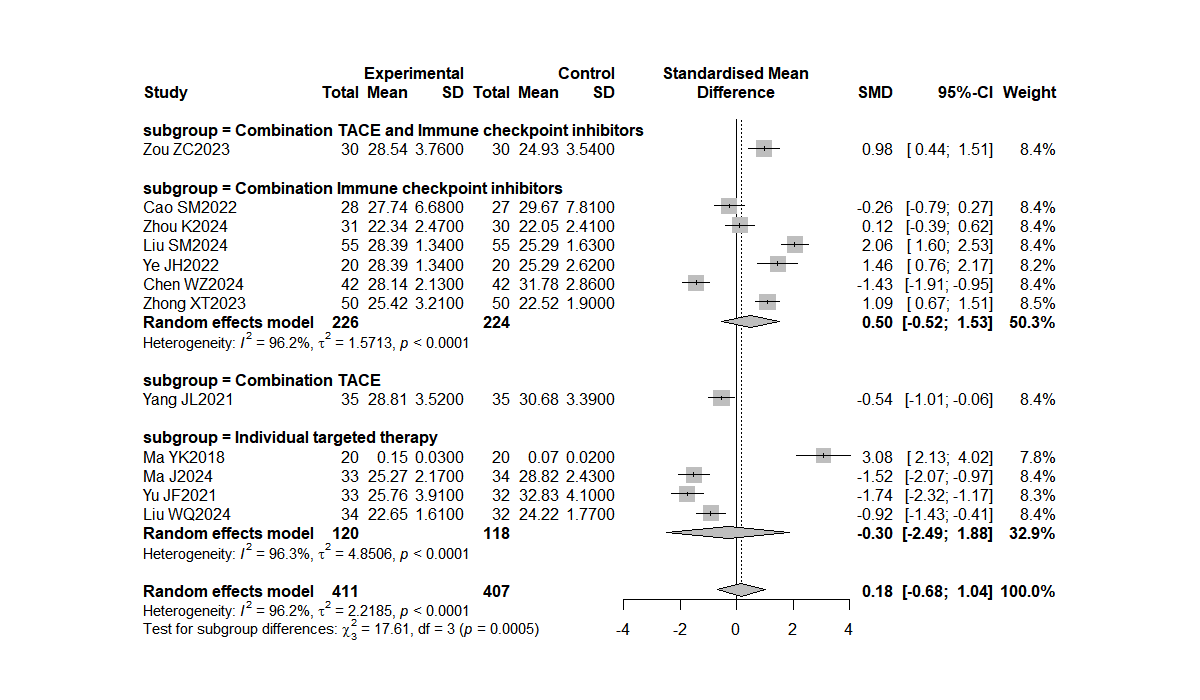


**Supplementary Figure 13:** Subgroup analysis of CD4^+^/CD8^+^.


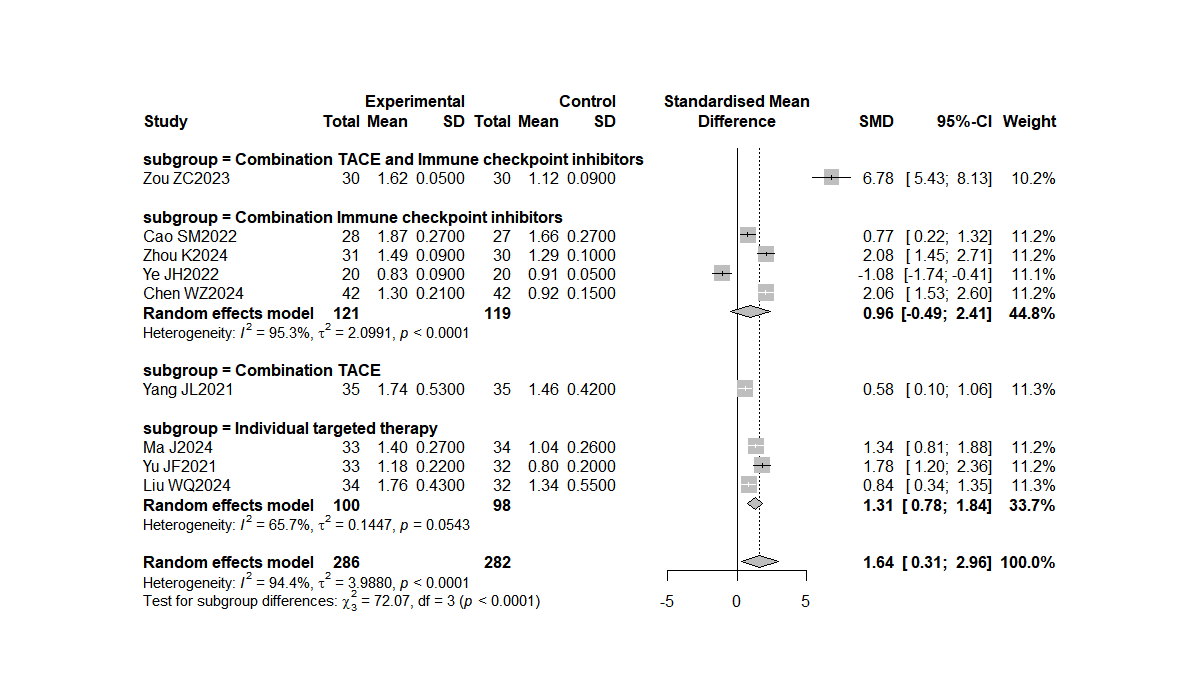


**Supplementary Figure 14:** Subgroup analysis of NK.


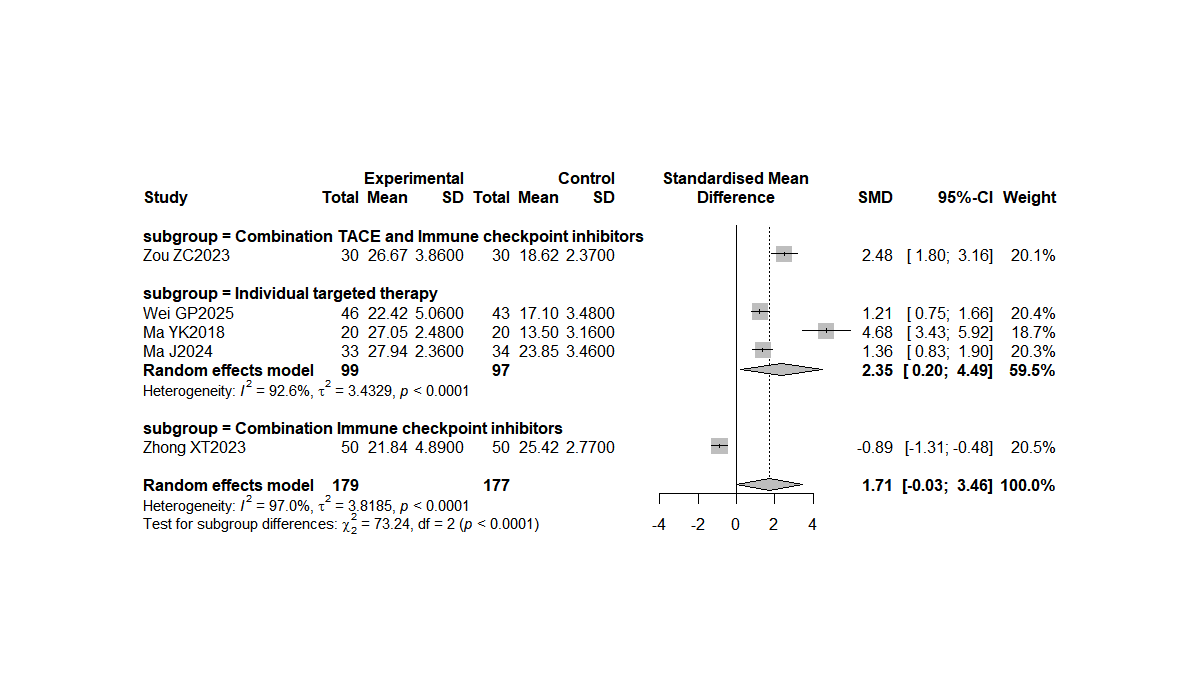


**Supplementary Figure 15:** Subgroup analysis of weakness.


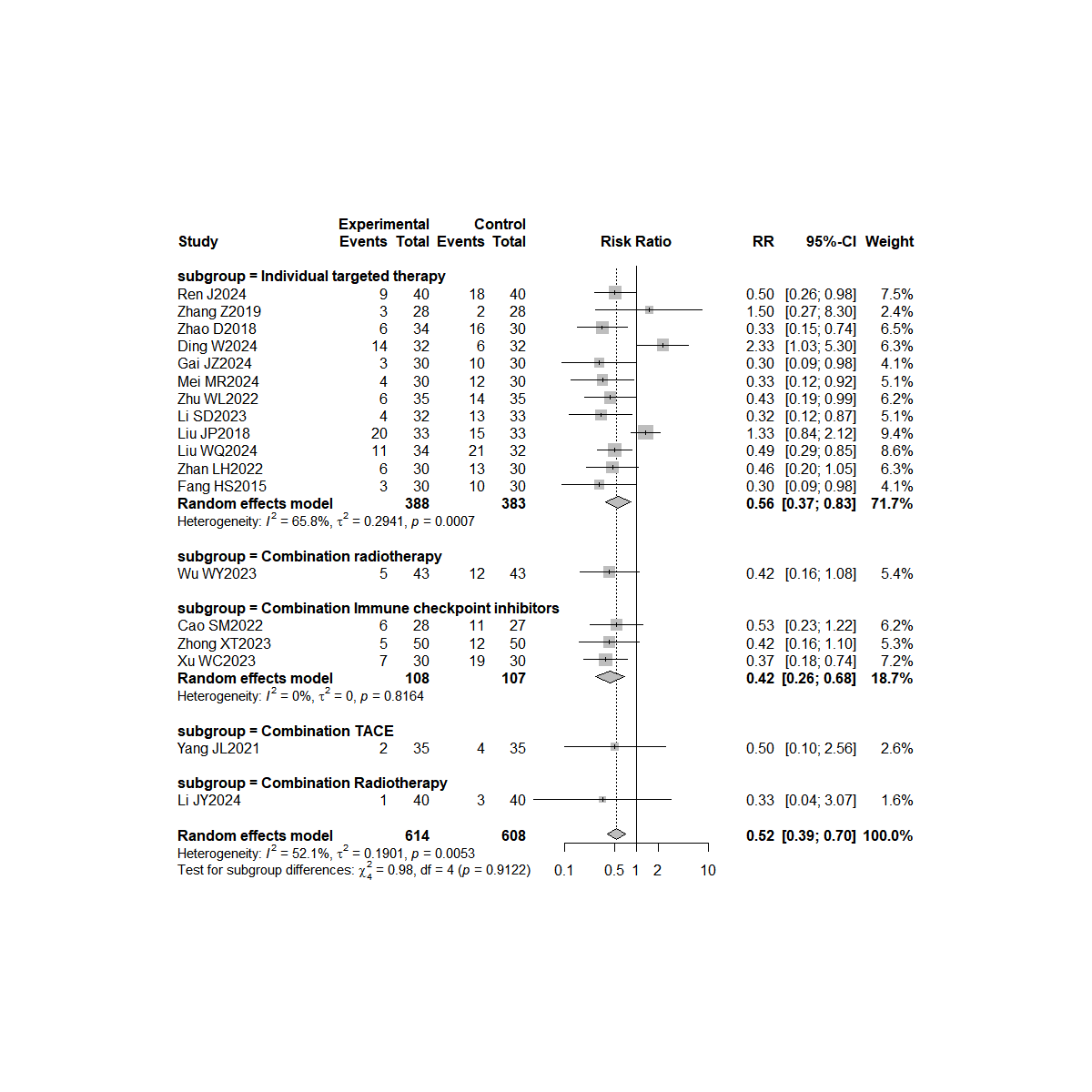


**Supplementary Figure 16:** Subgroup analysis of loss of appetite.


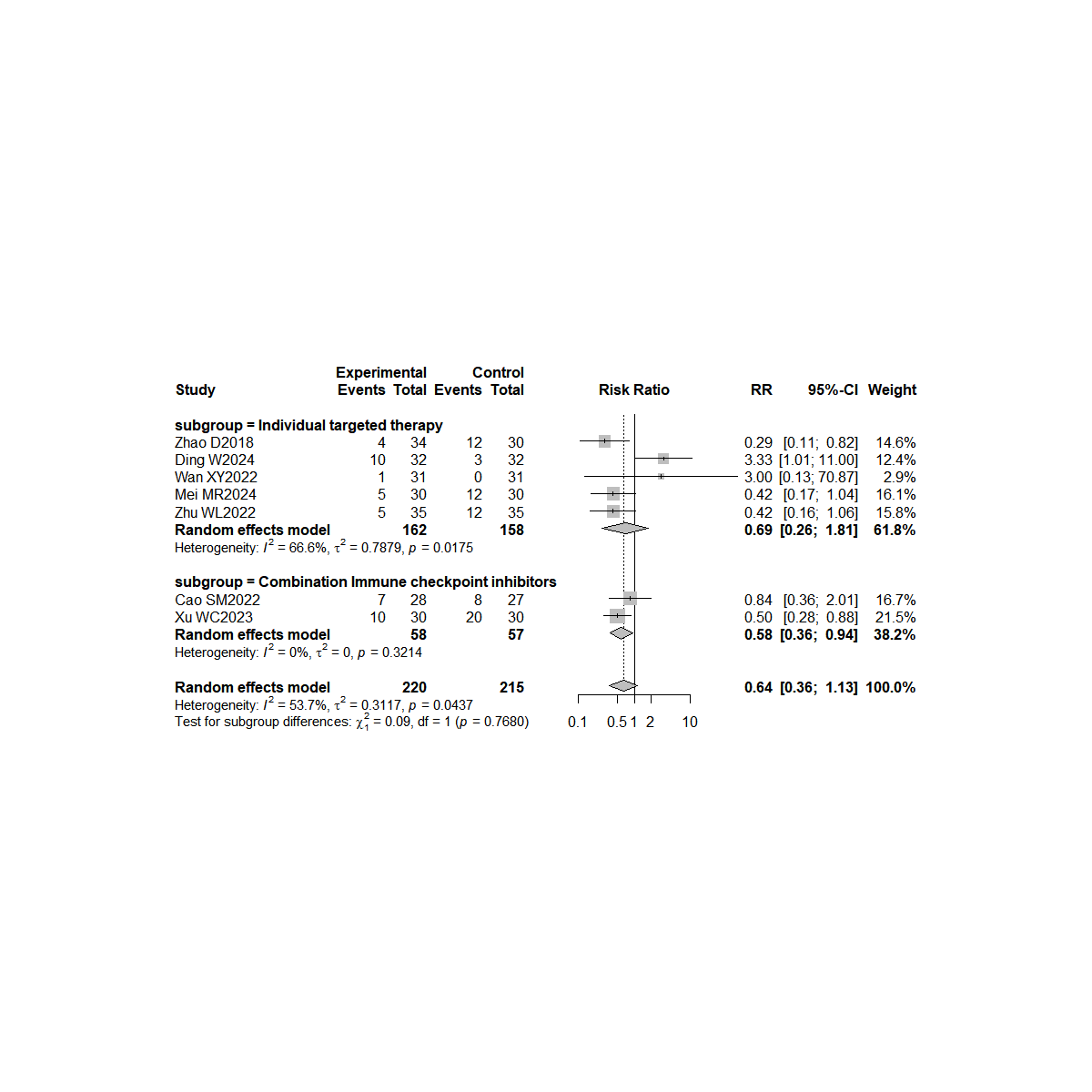


**Supplementary Figure 17:** Subgroup analysis of diarrhea.


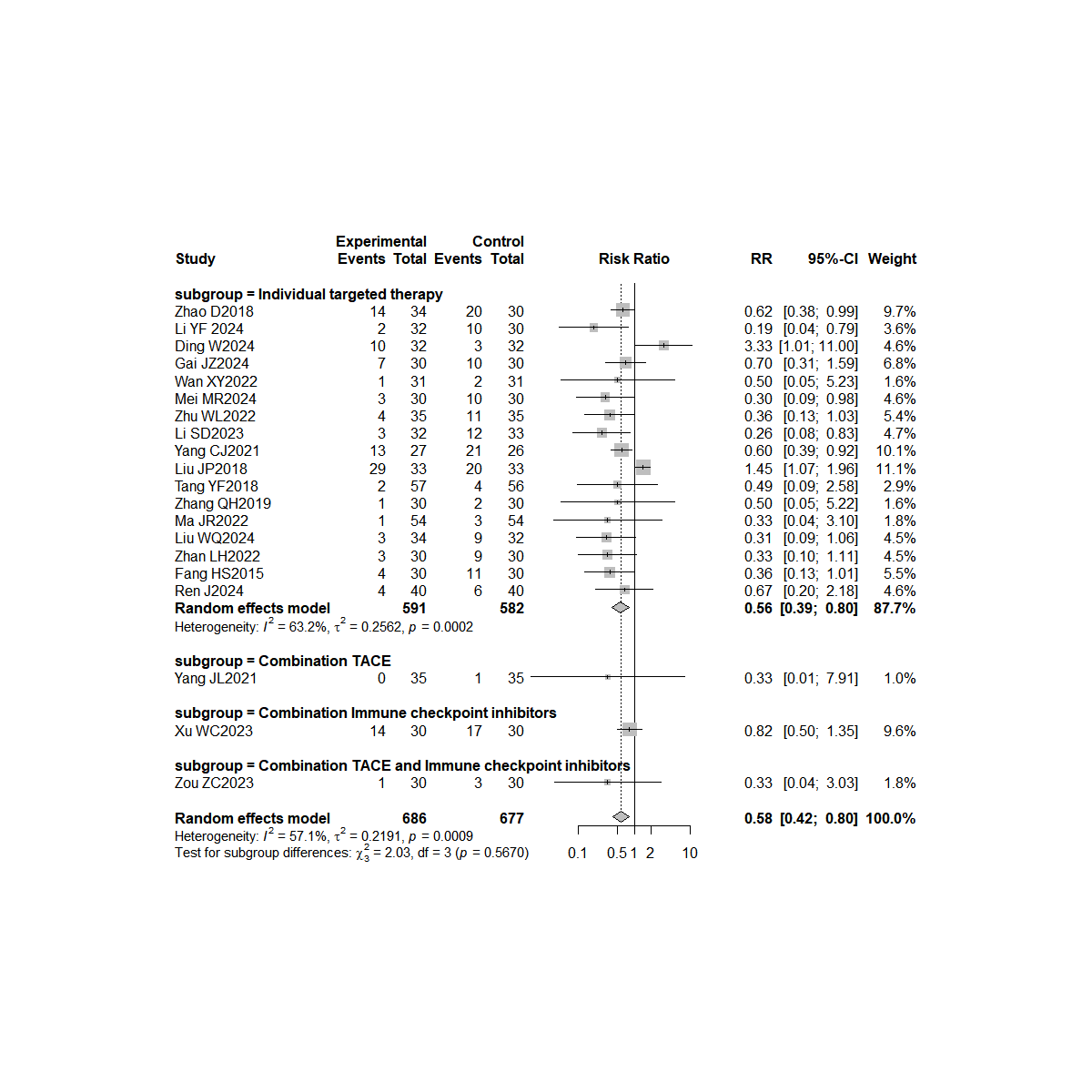


**Supplementary Figure 18:** Subgroup analysis of Chinese medicine evidence efficacy score.


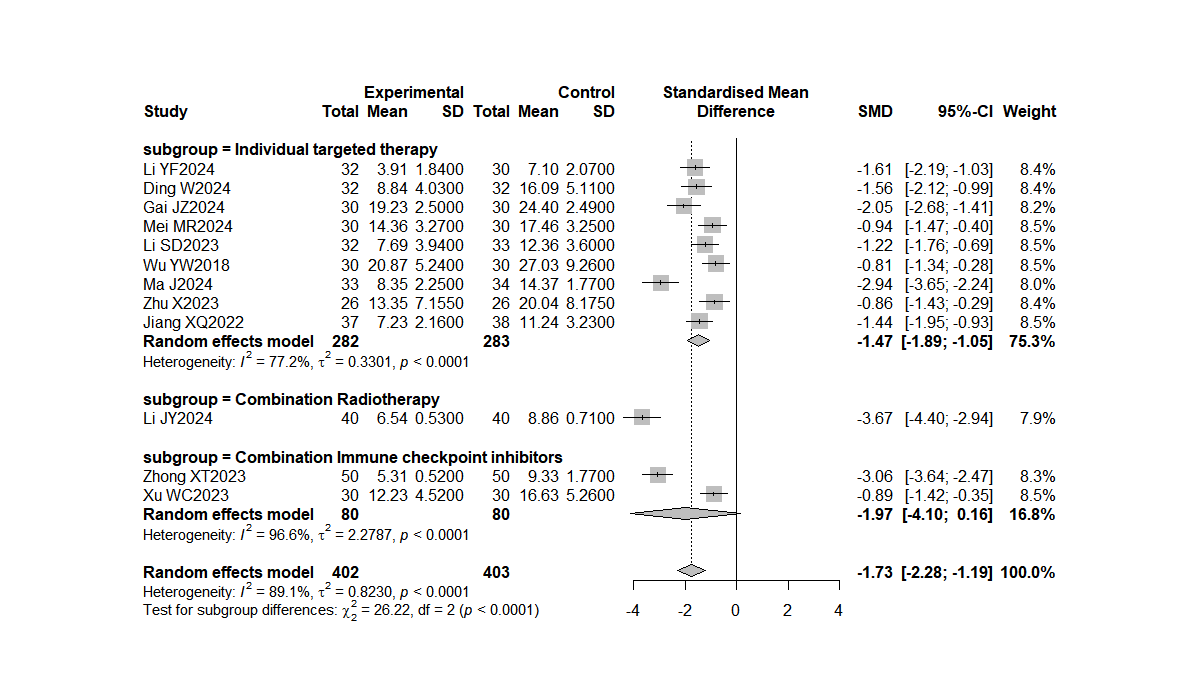


**Supplementary Figure 19:** Sensitivity analysis.


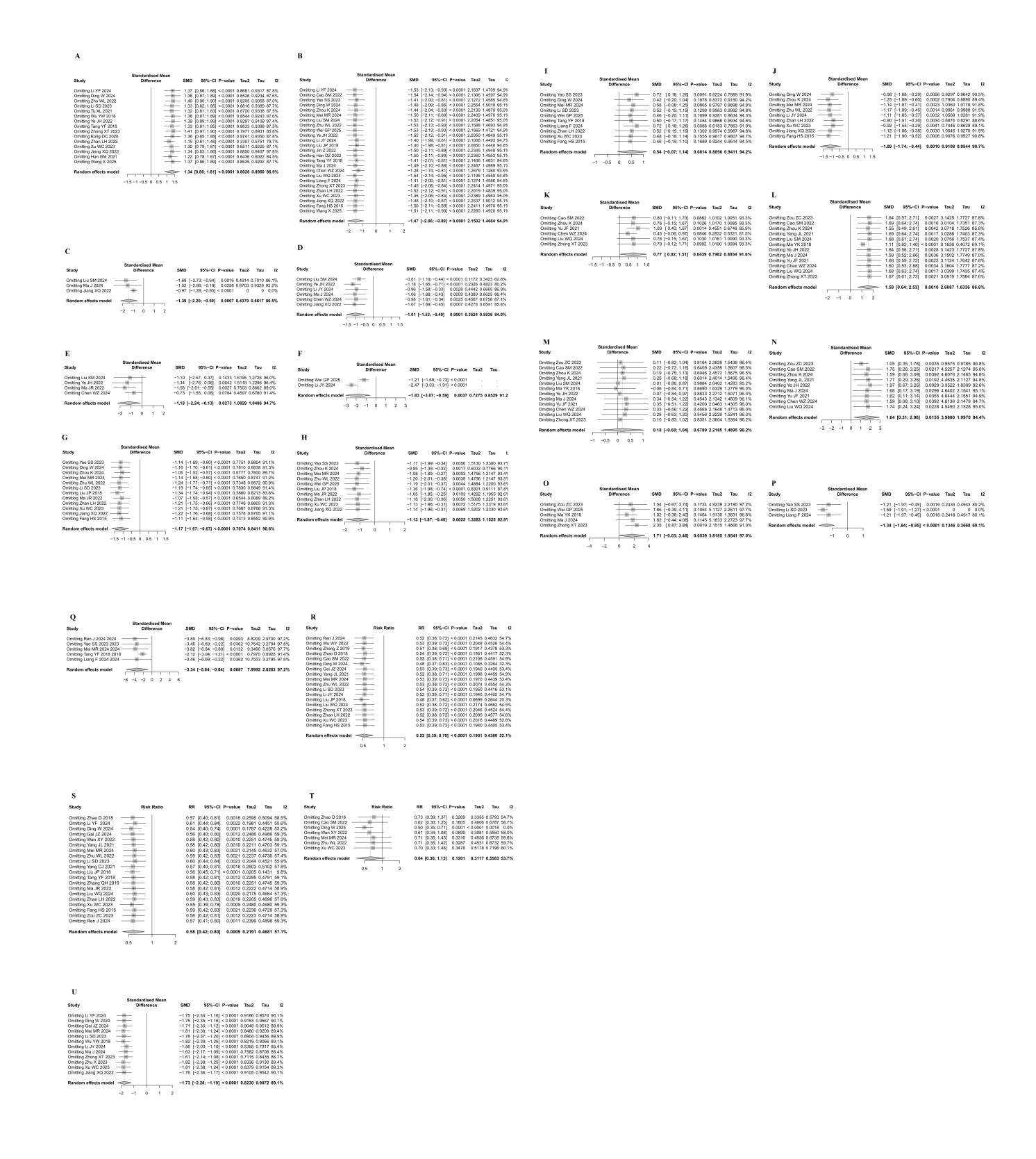

Supplement: Supplementary file 1 [file DataSheet1.docx]
